# Safe dosage and potential risks of chlorogenic acid: insights from *in vitro* and *in vivo* studies

**DOI:** 10.3389/fphar.2026.1740609

**Published:** 2026-02-24

**Authors:** Yilin Pang, Mengyao Jiang, Binjie Ge, Lin Huang, Xiaoyu Rao, Xueqing Wang, Huaibin Zhou, Jianxin Lyu, Zheng Wang, Guoqiang Tan

**Affiliations:** 1 Zhejiang Provincial Key Laboratory of Medical Genetics, Key Laboratory of Laboratory Medicine, Ministry of Education, China, School of Laboratory Medicine and Life Sciences, Wenzhou Medical University, Wenzhou, Zhejiang, China; 2 College of Bioscience and Biotechnology, Hunan Agricultural University, Changsha, Hunan, China; 3 Hunan Animal Pharmaceutical Company, Hunan New Wellful Company Limited, Hunan Agricultural Group Company, Hunan Agricultural Development & Investment Group Company, Changsha, Hunan, China; 4 The Affiliated Nanhua Hospital, Department of Laboratory Medicine, Hengyang Medical School, University of South China, Hengyang, Hunan, China; 5 State Key Laboratory of Genetic Engineering, School of Life Sciences, Fudan University, Shanghai, China; 6 Interdisciplinary Research Center on Biology and Chemistry, Shanghai Institute of Organic Chemistry, Chinese Academy of Sciences, Shanghai, China; 7 Laboratory Medicine Center, Department of Clinical Laboratory, People’s Hospital of Hangzhou Medical College, Hangzhou, China

**Keywords:** acute toxicity testing, chlorogenic acid, hepatocellular injury, hepatocyte protection, iron deficiency, polyphenol

## Abstract

**Introduction:**

As the economy grows, there is a growing emphasis on food safety. While the health benefits of chlorogenic acid (CGA) are recognized, safe dosages and potential liver cell damage from excessive CGA consumption are not well studied. This study aims to determine the safe and effective dose range of CGA and understand how it causes toxicity in hepatocyte at half-maximal inhibitory concentration (IC50).

**Methods:**

This study assessed the impact of various CGA concentrations on liver cells, examining growth, viability, toxicity, energy metabolism, and colony formation using Real-Time Cell Analysis (RTCA), CCK-8, lactate dehydrogenase (LDH) assays, and Seahorse XF96. It established CGA’s IC50 for cell viability and identified differentially expressed proteins via proteomics. Subsequently, Gene Ontology (GO) and Kyoto Encyclopedia of Genes and Genomes (KEGG) pathway analyses were conducted to elucidate the signaling pathways associated with the differentially expressed proteins. Further validation of the molecular mechanisms was performed using flow cytometry, Western blotting, and reverse transcription quantitative polymerase chain reaction (RT-qPCR). Finally, CGA was injected into Kunming (KM) mice via the tail vein for acute toxicity testing.

**Results:**

In this study, 200 µM of CGA significantly reduced LDH release and increased the mitochondrial oxygen consumption rate (OCR) in hepatocytes, but it did not affect the extracellular acidification rate (ECAR). Additionally, 200 µM of CGA slightly promoted hepatocyte growth; however, at 300 μM, CGA nearly completely inhibited the clonogenic capacity of hepatocytes, and at 600 μM, it significantly impeded hepatocyte growth. The IC50 of CGA for hepatocyte activity was determined to be 613.1 µM. *In vitro* experiments indicated that incubation with CGA at its IC50 concentration for 96 h resulted in the arrest of L-02 cells in the S phase of the cell cycle and induced apoptosis. Further investigation revealed that the IC50 concentration of CGA, through the depletion of free iron within hepatocytes, significantly reduced the expression of iron-sulfur cluster subunits in mitochondrial complexes I-III and disrupted the oxidative-reductive homeostasis of hepatocytes, ultimately leading to hepatotoxicity. Interestingly, N-Acetyl-L-cysteine (NAC) or ferric citrate reduced hepatocyte toxicity from excessive CGA. All mice survived after receiving CGA injections at doses up to 125 mg/kg. The semi-lethal concentration (LD_50_) for Kunming mice was 382.28 mg/kg.

**Conclusion:**

These findings suggest that the antioxidant and iron-chelating properties of CGA determine its role in either liver protection or toxicity at varying concentrations, providing valuable insight for its rational dietary and clinical use.

## 1 Introduction

Chlorogenic acid (CGA), also called caffeoylquinic acid (CQA), is a dietary polyphenol formed by the esterification of caffeic acid (CA) and quinic acid (QA) (Pang et al., 2022; Huang et al., 2015; Liu et al., 2015). It is commonly found in the human diet and exhibits various biological effects, including anti-inflammatory, antioxidant, antiviral, hypoglycemic, lipid-lowering, anti-tumor, and liver-protective properties (Ji et al., 2013; Catanzaro et al., 2016; Yamagata et al., 2018). CGA at a concentration of 40 μg/mL (112.90 µM) clears over 70% of DPPH (1,1-diphenyl-2-picrylhydrazyl radical) free radicals *in vitro*, and at 25 μM, it reduces reactive oxygen species (ROS) to alleviate oxidative stress in intestinal cells (Song et al., 2024). CGA is the main active ingredient in 61 Chinese medicinal herbs and various fruits and vegetables, such as *eucommia*, *honeysuckle*, and *green tea* (Ru et al., 2014). It is widely used across the food, chemical, medicine, and healthcare industries. Additionally, CGA has been recognized as a natural anticancer drug and has gained approval from the China Food and Drug Administration (Gupta et al., 2022).

The liver is vital for metabolism, the endocrine regulation, and immune response but is often exposed to irritants like viruses, medications, and chemicals (Wang et al., 2024). CGA has the ability to mitigate inflammation and oxidative damage by modulating various signaling pathways, thereby ameliorating liver injury caused by these irritants (Park et al., 2015; Yao et al., 2025; Xu et al., 2025). Specifically, CGA mitigates liver injury and intrahepatic cholestasis caused by α-naphthylisothiocyanate (ANIT) by inhibiting the STAT3 (signal transducer and activator of transcription 3) and NF-κB pathways (Tan et al., 2016). Wang Y. C. et al. (2017) showed that CGA can reduce bleomycin-induced pulmonary fibrosis by inhibiting endoplasmic reticulum stress. Lee et al. (2017) found that CGA derivatives increase caveolin-1 expression and suppress the EGFR (Epidermal growth factor receptor)/STAT3/iNOS (inducible nitric oxide synthase) pathway, reducing oxidative stress and alcoholic fatty liver in mice. Wei et al. (2018) demonstrated that CGA protects against acetaminophen (APAP)-induced liver toxicity by activating the ERK/Nrf2 (nuclear factor erythroid 2-related factor 2) antioxidative pathway in mice and hepatocytes. Additionally, their study showed that CGA aids liver regeneration in APAP-intoxicated mice by activating early growth response-1 (EGR1) transcription (Wei et al., 2023).

**FIGURE 1:**
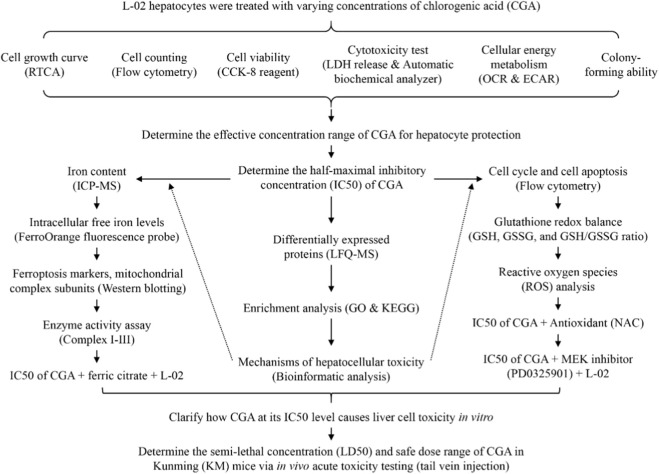
The study flow chart.

Building on the aforementioned research findings, we propose the hypothesis that an optimal concentration of CGA confers protection to liver cells via its antioxidant properties. Conversely, excessive levels of CGA, upon oxidation, may induce significant hepatotoxicity. Consequently, the aim of this study is to delineate the safe and efficacious dosage range of CGA and to elucidate its toxicological mechanisms in hepatocytes at the IC50 concentration. To address these questions, this study systematically assessed how various concentrations of CGA impact liver cell growth, viability, toxicity, energy metabolism, and colony formation. Additionally, the research determined the semi-lethal concentration (LD50) and established the safe dosage range of CGA in Kunming (KM) mice through *in vivo* acute toxicity testing via tail vein injection. The detailed methodological framework is presented in Figure 1. By employing this methodology, the study aims to provide a theoretical basis for the judicious application of CGA.

## 2 Materials and methods

### 2.1 Cell lines and cell culture

L-02 hepatocytes from the Cell Bank of the Chinese Academy of Sciences (Shanghai, China) were cultured in Dulbecco’s Modified Eagle Medium (DMEM) (Gibco, Grand Island, NY, United States) with 10% fetal bovine serum (FBS; Gibco) and antibiotics (100 U/mL of penicillin and 100 μg/mL of streptomycin) in a 5% CO_2_ incubator at 37 °C. Cell lines were confirmed mycoplasma-free before experiments.

### 2.2 Reagents, antibodies and chemicals

CGA was obtained from Alfabiotech (Chengdu, China). Antibodies against PARP (ab191217), Caspase-9 (ab32539), Cyclin D1 (ab40754), CDK6 (ab124821), p21 (ab109520), Cyclin A2 (ab32386), Cyclin E1 (ab33911), ASNS (ab40850), GPX4 (ab125066), Ferritin (ab75973), TfR1 (ab214039), IREB2 (ab232994), RRM2 (ab57653), c-Myc (ab32072), NDUFV2 (ab183715), NDUFS3 (ab177471), NDUFS8 (ab170936), Grim19 (ab110240), SDHA (ab137040), SDHB (ab178423), UQCRB (ab190360), UQCRC2 (ab203832), UQCRFS1 (ab191079), COX IV (ab202554), ATP5A (ab176569), and TOMM20 (ab186735) were obtained from Abcam (Cambridge, MA). Antibodies against GAPDH (2118S), p-Erk1/2 (4370T), and SLC7A11 (32683S) were bought from Cell Signalling Technology (Danvers, MA). β-actin antibody (AF0003), MTT kit, and CCK-8 kit were purchased from Beyotime Biotechnology (Shanghai, China). MitoSOX red and Carboxy-H_2_DCFDA were obtained from Thermo Fisher Scientific (Waltham, MA). SB203581, SP600125, and PD0325901 were purchased from Selleck Chemicals (Houston, TX, United States).

### 2.3 Real-time cell analysis (RTCA), lactate dehydrogenase (LDH) release, cytotoxic and label-free quantitative mass spectrometry (LFQ-MS) assays

The RTCA operational procedure was described as previously mentioned (Lan et al., 2018). Briefly, the effect of different CGA concentrations (0, 200, 400, and 800 μM) on L-02 hepatocyte growth was assessed using the RTCA system. Cells were seeded at 2 × 10^3^ per well in a 10-well plate with a microelectronic biosensor chip, similar in size to a 96-well plate. After overnight incubation at 37 °C, the medium was replaced with DMEM containing various CGA concentrations. Cell growth was monitored continuously for 100 h using the RTCA system. The cell index, indicating changes in electrical impedance as a measure of cell migration, was calculated with RTCA Software Package 2.0. Each sample was tested in triplicate across three experiments.

LDH cytotoxicity assay was performed as previously described (Ma et al., 2022). Briefly, L-02 cells were seeded at 2 × 10^3^ cells per well in 96-well plates. After 24 h at 37 °C, the medium was replaced with fresh DMEM containing 1% FBS and varying CGA concentrations. The wells were categorized into cell-free, untreated control, untreated control for cell lysis (serving as a control for maximum enzyme activity), and CGA-treated groups. The cells incubated at 37 °C for 48 h, followed by the addition of 100 μL of the same medium and another 47-h incubation. LDH release reagent was then added, and after 96 h total incubation, plates were centrifuged at 400 *g* for 5 min. Finally, 120 μL of supernatant from each well was transferred to a new plate for analysis using an LDH Cytotoxicity Assay Kit (Beyotime Biotechnology, Shanghai, China). Absorbance at 490 nm was measured for each well using a microplate reader (Thermo Fisher Scientific, Waltham, MA), with 600 nm as the reference wavelength. The data were then plotted into bar charts using GraphPad Prism 5.

Cytotoxicity assays and LFQ-MS were performed according to established protocols (Pang et al., 2022). Raw mass spectrometry files were analyzed using Protein Discoverer (v1.4.0.288) and Mascot (v2.3.2).

### 2.4 Automatic biochemical analyzer assay

LDH and alkaline phosphatase (ALP) levels were measured using the rate method on a Siemens ADVIA2400 Automatic Biochemical Analyzer. All procedures were performed in strict adherence to the manufacturer’s instructions for the Siemens original reagents. The data were then plotted into bar charts using GraphPad Prism 5.

### 2.5 Quantification of oxidative phosphorylation and glycolysis

The Seahorse XF96 Extracellular Flux Analyzer (Seahorse Bioscience, North Billerica, MA, United States) was utilized to measure real-time integrated cellular oxygen consumption rate (OCR) and extracellular acidification rate (ECAR) as previously outlined (Lan et al., 2018; Wang L. et al., 2017). Data is presented using Agilent’s Wave Desktop software (Agilent Technologies Inc., SC, CA, United States).

### 2.6 Western blotting (WB), real-time quantitative PCR (RT-qPCR) and reactive oxygen species (ROS) analysis

WB analysis, RT-qPCR, and ROS analysis were performed as previously described (Pang et al., 2022). Primers for RT-qPCR were synthesized by BGI (Shenzhen, China) and are listed in Supplementary Table S1. Data processing and presentation were conducted using BioRadCFXManager 3.0 (Bio-Rad, Hercules, CA) and flow cytometry software from ACEA Biosciences, United States.

### 2.7 Preparation of mitochondria and assay of enzyme activity

The enzyme activity of three respiratory-chain complexes was quantified in the mitochondria of L-02 hepatocytes treated with CGA and in untreated cells, as previously described (Birch-Machin and Turnbull, 2001; Bao et al., 2018; Liu et al., 2018). Data is acquired and displayed using the Hitachi U3900 UV-vis spectrometer and GraphPad Prism 5.

### 2.8 Quantitative analysis of oxidized and reduced forms of glutathione (GSH)

To detect GSH and GSSG (glutathione disulfide), two 200 μL sample solutions were prepared. L-02 hepatocytes, with or without 600 μM CGA treatment for 96 h, were digested, centrifuged at 4 °C and 1,000 rpm for 4 min, and washed with PBS (phosphate buffered saline). The cell concentration was adjusted to 1 × 10^7^ cells/ml, centrifuged again, and lysed with 80 μL of 10 mM HCl. After two freeze-thaw cycles, 20 μL of 5% 5-sulfosalicylic acid (SSA) was added, followed by centrifugation at 8,000×g for 10 min. The supernatant was transferred, diluted with ddH_2_O, and SSA concentration adjusted to 0.5%. GSH and GSSG levels were measured using the GSSG/GSH Quantification Kit from Dojindo Laboratories, Japan, following the manufacturer’s instructions. The data were then plotted into bar charts using GraphPad Prism 5.

### 2.9 Inductively coupled plasma mass spectrometry (ICP-MS) assay

L-02 cells were cultured in 10 cm dishes at an initial density of 4 × 10^5^ cells per dish and treated with either 0 or 600 μM CGA. After 96 h, the cells were harvested using pancreatic enzymes, washed twice with PBS, and lysed with 1% TritonX-100 (300 μL for 0 μM CGA and 150 μL for 600 μM CGA) on ice for 10 min. The lysates were centrifuged at 12,000 g and 4 °C for 10 min. A 2 μL sample was used for protein concentration analysis with a BCA assay kit (Thermo Fisher Scientific, Waltham, MA). The remaining sample was resuspended in 5% nitric acid (5 mL) and digested under high pressure. The iron content in the digested sample was then measured using ICP-MS (Agilent) according to the manufacturer’s guidelines and performed as previously described (Ren et al., 2021). The data were then plotted into bar charts using GraphPad Prism 5.

### 2.10 Acute toxicity testing

Fifty Kunming (KM) mice (17–20 g, equal male and female) were obtained from Hunan Slack Jingda Experimental Animal Co., LTD. After a week of acclimatization, they were divided into five groups of 10 mice each, maintaining equal gender distribution. Following a 16 h fast, each group received a single injection of CGA at different doses (normal saline, 125 mg/kg, 250 mg/kg, 375 mg/kg, and 500 mg/kg). The 7-day mortality rate and daily mouse weights were tracked, and the LD_50_ of CGA injection was determined as previously described (Schlede et al., 1992).

Blood was drawn from the eyeballs, incubated at 37 °C for 2 h, and then refrigerated at 4 °C overnight. The next day, the serum was separated by cryocentrifugation at 2000 rotations/min for 10 min and transferred to a sterile centrifuge tube. The serum was diluted with SA buffer (about 300 μL) and analyzed for biochemical indices using the Hitachi 7,060 automatic analyzer, measuring AST, ALT, ALP, T-Bil-V2, UREA, and CREA-S. The data were then plotted into bar charts using GraphPad Prism 5.

### 2.11 Statistical analysis

Data that follow a normal distribution are presented as the mean ± standard deviation (SD). Comparisons between two groups were performed using an independent Student’s *t*-test. For comparisons involving three or more groups with a single variable, a one-way ANOVA was employed. When comparing three or more groups with two variables, a two-way ANOVA was utilized. For measurement data not conforming to a normal distribution, comparisons between two groups were conducted using the Mann-Whitney test. For comparisons involving three or more groups with a single variable, the Kruskal-Wallis test was applied. All analyses were conducted using GraphPad Prism 5 software. Statistical significance was determined at a *p*-value of less than 0.05.

## 3 Results

### 3.1 Hepatocytes can tolerate high levels of CGA, and the appropriate amount of CGA can effectively protect hepatocytes

**FIGURE 2:**
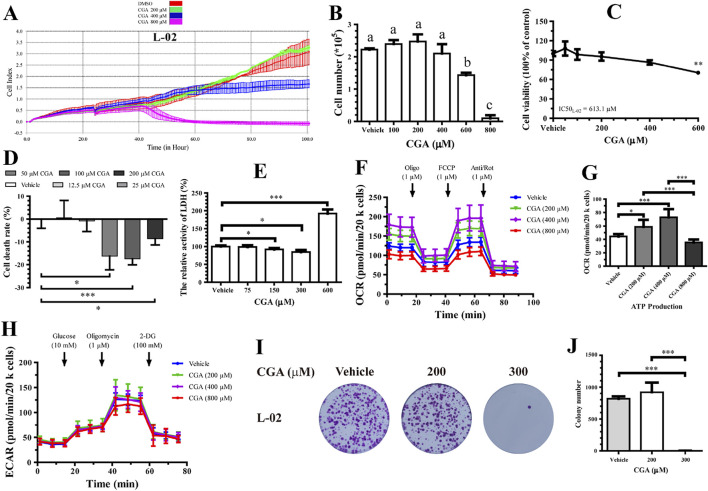
Adequate levels of chlorogenic acid (CGA) protected human hepatocytes, but too much caused harm and inhibited growth *in vitro*. **(A)** Utilized Real-Time Cell Analysis (RTCA) to investigate the impact of CGA on the growth kinetics of L-02 hepatocytes. The initial inoculation density of L-02 cells was 2 × 10^3^ cells per well. **(B,C)** L-02 cells were treated with different levels of CGA for 96 h. Cells were placed in 12-well plates at a density of 2 × 10^4^ cells per well. Cell viability was measured using flow cytometry and the CCK-8 reagent. Results show mean ± standard deviation, with the IC50 of CGA was determined and is reported in **(C)**. **(D)** The impact of CGA on L-02 cell toxicity was measured using the Lactate Dehydrogenase (LDH) Cytotoxicity Assay Kit after exposing the cells to different CGA concentrations for 96 h in DMEM medium with 1% FBS. **(E)** Hepatocytes were treated with either a vehicle or varying levels of CGA for 96 h in medium with 5% FBS. LDH levels in the supernatant were measured using an Automatic Biochemical Analyzer and normalized to cell lysate protein concentration. **(F)** L-02 (2 × 10^4^ cells/well) cells were treated with different concentrations of CGA for 4 h in DMEM medium with 10% FBS. The effect of CGA on mitochondrial oxygen consumption rate (OCR) was measured in real-time using the Seahorse XF96 Extracellular Flux Analyzer. Basal OCR levels were recorded at three time points, after which the ATP synthase inhibitor oligomycin (1 µM), uncoupler FCCP (1 µM), complex I inhibitor rotenone (1 µM), and complex III inhibitor antimycin A (1 µM) were sequentially injected. **(G)** The impact of CGA on ATP production in L-02 cells (n = 6). **(H)** The cellular aerobic glycolysis was assessed through measurement of the extracellular acidification rate (ECAR) in L-02 cells (2 × 10^4^ cells/well) treated with varying concentrations of CGA (0, 200, 400, 800 μM) in DMEM medium containing 10% FBS for a duration of 4 h. The injection order of glucose (10 mM), oligomycin (1 μM), and 2-DG (100 mM) was followed for the L-02 cells (n = 6). **(I)** The impact of CGA on the colony-forming ability of L-02 cells. **(J)** Colony numbers were quantified using ImageJ Plus software, with the results expressed as a percentage of the control group. The data are presented as the mean ± standard deviation (n = 3). *p < 0.05, **p < 0.01, ***p < 0.001. ^a–c^ Mean values not sharing a common superscript were significantly different among the groups (*p* < 0.05).

Figures 2A–C show how RTCA, flow cytometry, and CCK-8 (Cell Counting Kit-8) were used together to analyze the growth and dose-response curves of hepatocytes L-02 treated with different concentrations of CGA. The results suggest that concentrations above 400 μM inhibit hepatocyte proliferation after 96 h, with 800 μM showing toxicity. Concentrations between 100 and 200 μM have a slight stimulatory effect on proliferation, but this effect is not statistically significant. The IC50 of CGA for hepatocyte activity was determined to be 613.1 µM (Figure 2C). For subsequent mechanistic experiments, we used 600 µM as a working approximation of the IC50 concentration. LDH activity in the L-02 cell medium was measured after incubation with different concentrations of CGA and FBS for 96 h using a LDH Cytotoxicity Assay Kit and a biochemical analyzer. Lower CGA concentrations (50–300 μM) decreased LDH activity in L-02 cells (*p* < 0.05), suggesting a positive effect. However, higher concentrations (600 μM) led to increased LDH activity (*p* < 0.05), indicating hepatocyte toxicity (Figures 2D,E).

In Figure 2F, hepatocyte OCR decreased with oligomycin treatment and increased with FCCP (a mitochondrial oxidative phosphorylation uncoupler) treatment. Hepatocytes treated with 200 and 400 µM of CGA showed a significant increase in OCR compared to controls, with a concentration-dependent effect observed between 200 and 400 µM. Figure 2G and Supplementary Figure S1A-C show that hepatocytes had increased basal, maximal, spare respiration, and intracellular ATP production after a 4 h incubation with 200 μM and 400 µM CGA (*p* < 0.05). However, 800 µM CGA did not have a significant effect on these mitochondrial energy metabolism indicators in hepatocytes. Hepatocytes treated with 800 µM CGA exhibited decreased OCR compared to the control group. ECAR values for hepatocytes treated with 200–800 µM CGA for 4 h showed no change in glycolysis (Figure 2H). Incubation with 200 µM CGA for 15 days did not significantly impact the clonogenic ability of L-02 cells; however, treatment with 300 µM CGA almost completely inhibited L-02 cell clonogenesis (*p* < 0.001) (Figures 2I,J).

### 3.2 Proteomics was used to study how the IC50 of CGA affects hepatocytes at a molecular level

**FIGURE 3:**
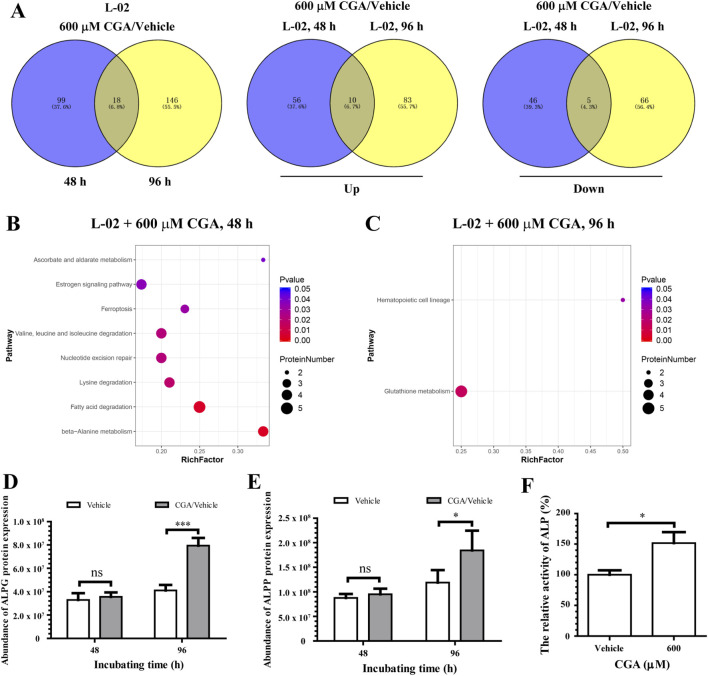
Utilized label-free quantitative mass spectrometry to identify novel targets of L-02 cells with an IC50 of CGA, in order to establish a theoretical basis for understanding the hepatotoxic effects of excessive CGA. **(A)** Venn diagrams were utilized to illustrate the differential expression of proteins observed in comparisons of samples incubated with CGA (600 μM) for varying durations. The number of up-regulated and down-regulated proteins in each group comparison was noted, with “Down” indicating downregulated proteins and “Up” indicating upregulated proteins. **(B,C)** Enriched KEGG pathways were identified for proteins that exhibited differential expression with a T-test value less than 0.05. The horizontal axis denotes the rich factor, which represents the proportion of differentially expressed proteins within a given Gene Ontology (GO) term relative to the total number of annotated proteins within that term. The designation of 600 μM CGA/Vehicle signifies the average differences in protein expression between each group treated with CGA compared to the untreated group (n = 3). **(D,E)** The impact of CGA on the intensities of ALPG **(D)** and ALPP **(E)** was quantitatively assessed using label-free mass spectrometry. **(F)** L-02 cells were exposed to either vehicle or CGA (600 μM) in DMEM medium containing 5% FBS for a duration of 96 h. The levels of alkaline phosphatase (ALP) in the medium supernatant were assessed using an automatic biochemical analyzer, with the activity normalized to the protein concentration of the cell lysate. The results are expressed as mean values ±standard deviation (n = 3). ns, not significant; *p < 0.05, ***p < 0.001.

Figure 3A shows proteomic screening results: 117 differential proteins identified after 48 h and 164 after 96 h of incubation with 600 µM CGA. Venn diagram analysis identified a total of 18 overlapping differential proteins between the two groups, including 10 upregulated and 5 downregulated proteins. The bioinformatics analysis suggests that dysregulation of multiple signaling pathways, such as glutathione metabolism, beta-alanine metabolism, fatty acid degradation, lysine degradation, nucleotide excision repair, valine, leucine, and isoleucine degradation, and ferroptosis, may contribute to hepatocyte toxicity at the IC50 of CGA (Figures 3B,C). Proteomic analysis incidentally revealed alterations in the expression of alkaline phosphatase isozymes (ALPG and ALPP) (Figures 3D,E), and a corresponding increase in ALP activity was detected in the culture supernatant (Figure 3F).

### 3.3 CGA overdosage resulted in the notable induction of hepatocyte cell cycle arrest and apoptosis

**FIGURE 4:**
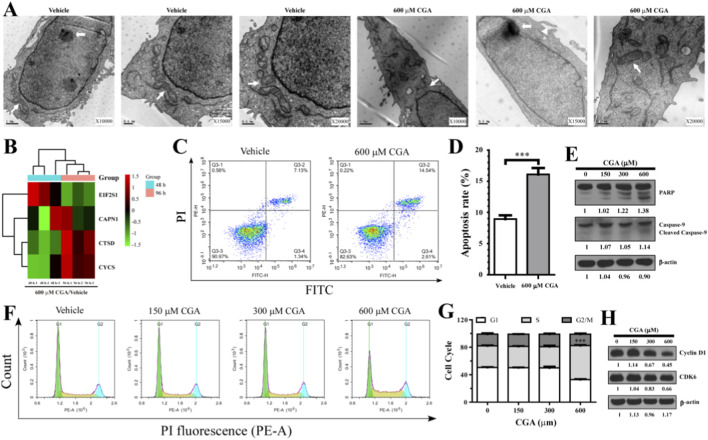
The IC50 of CGA led to the arrest of L-02 cells at the S phase of the cell cycle and triggered apoptosis. **(A)** The morphological alterations of hepatocytes were observed using transmission electron microscopy (TEM) under conditions of both CGA overdose and absence. The nucleus is denoted by thick white arrows, while the mitochondria are indicated by thin white arrows. **(B)** A heat map was generated to display the hierarchical clustering of apoptosis-related proteins that exhibited differential expression at two distinct time points following treatment with CGA. **(C,D)** The induction of apoptosis in L-02 cells by CGA was assessed using flow cytometry **(C)**, and the resulting apoptosis rates of CGA-treated L-02 cells are presented in **(D)**. **(E)** Western blot analysis was conducted on apoptosis-related proteins (PARP, 113 kDa; Caspase-9, 46 kDa) in L-02 cells that were exposed to varying concentrations of CGA (0, 150, 300, and 600 μM) in DMEM medium supplemented with 5% FBS for a duration of 96 h (β-actin, 42 kDa). **(F)** Cell cycle distribution was assessed in response to varying concentrations of CGA (0, 150, 300, and 600 μM) over a 96-h period using flow cytometry. **(G)** Quantitative data obtained from **(F)** indicated the distribution of the cell cycle. **(H)** A Western blot analysis was conducted to investigate the impact of the IC50 of CGA on the expression levels of cell cycle-related proteins (Cyclin D1, 34 kDa; CDK6, 37 kDa) in L-02 cells. The data is expressed as means ± standard deviation (n = 3). ***p < 0.001.

As illustrated in Figure 4A, electron microscopy showed no major changes in the mitochondrial morphology of L-02 cells treated with 600 μM CGA; however, there were alterations in cell size and nucleolar morphology, which indicate early apoptosis. Proteomic analysis indicated a significant upregulation of CTSD (cathepsin D), CAPN1 (calpain 1), and CYCS (cytochrome c) proteins following treatment with 600 µM CGA (*p* < 0.05) (Figure 4B). Conversely, the same concentration of CGA resulted in a significant downregulation of EIF2S1 (eukaryotic translation initiation factor 2 subunit) protein (*p* < 0.05) (Figure 4B). Flow cytometry analysis showed that an IC50 of CGA induced apoptosis in hepatocytes, resulting in a 7.2% increase in apoptotic cells compared to the control group (*p* < 0.001) (Figures 4C,D). Western blot analysis revealed that CGA enhanced the cleavage of PARP and caspase-9 precursors in a dose-dependent manner (Figure 4E). Additionally, flow cytometry analysis indicated that the L-02 cell cycle was arrested in the S phase due to CGA treatment (*p* < 0.001) (Figures 4F,G). The findings from the Western blot analysis demonstrated that treatment with 600 µM CGA resulted in a significant decrease in the protein expression of Cyclin D1 and CDK6, which are involved in the G1/S phase transition (Figure 4H).

### 3.4 The key molecular mechanism of cytotoxicity is the regulation of high CGA concentration on iron metabolism and the glutathione redox balance in hepatocytes

**FIGURE 5:**
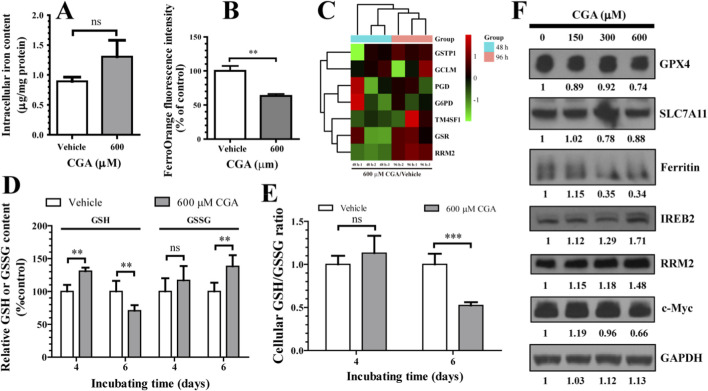
The observed toxicity at the IC50 of CGA can be attributed to the depletion of free iron within hepatocytes and the disruption of their oxidative-reductive homeostasis. **(A)** ICP-MS analysis was conducted to determine the total iron content in L-02 cells following a 96-h incubation period with CGA. **(B)** L-02 cells were treated with CGA for 96 h, then incubated with FerroOrange fluorescence probe (1 μM) for 30 min. Intracellular free iron levels were measured using an Automatic microplate reader. **(C)** A heat map was created to show the clustering of proteins involved in ferroptosis and glutathione metabolism, based on average differences in protein expression between groups. Data from three cell samples per group were used for this analysis. **(D)** A histogram depicting the relative levels of reduced and oxidized glutathione in L-02 cells following incubation with CGA at its IC50 for a duration of 4 or 6 days. **(E)** The GSH/GSSG ratio was measured in L-02 cells following treatment with or without CGA (600 μM) for a duration of 4 or 6 days. **(F)** The Western blot technique was utilized to analyze the expression of marker proteins associated with ferroptosis and iron metabolism (GPX4, 22 kDa; SLC7A11, 39 kDa; Ferritin, 21 kDa; IREB2, 105 kDa; RRM2, 45 kDa; c-Myc, 45 kDa) (GAPDH, 37 kDa). Data is shown as mean ± standard deviation (n = 3). ns, not significant; **p < 0.01, ***p < 0.001.

KEGG analysis showed a link between CGA and pathways involving glutathione metabolism, fatty acid degradation, and ferroptosis (Figures 3B,C). Initially, through the application of proteomics, Western blotting, and qRT-PCR, it was verified that treatment with CGA at a concentration of 600 µM resulted in an upregulation of transferrin receptor 1 (TfR1) expression and *Chac1* (glutathione-specific gamma-glutamylcyclotransferase 1) expression, as well as a downregulation of glutathione peroxidase 4 (GPX4) expression. All of these changes are associated with ferroptosis (Figure 5F; Supplementary Figure S2A,B). Furthermore, ICP-MS analysis indicated that the IC50 of CGA led to an increase in the total iron content within hepatocytes, although this increase was not statistically significant (Figure 5A). In contrast, the use of the FerroOrange probe demonstrated that the IC50 of CGA reduced the levels of free ferrous ions in hepatocytes (Figure 5B). Additionally, Western blot analysis revealed no significant change in the expression of SLC7A11 (cystine/glutamate transporter), a key protein in the ferroptosis pathway (Figure 5F).

Further investigation found that CGA increased the mRNA and/or protein levels of key enzymes involved in glutathione metabolism (*p* < 0.05), including RRM2 (ribonucleoside-diphosphate reductase subunit M2), GSR (glutathione reductase), GCLM (glutamate--cysteine ligase regulatory subunit), TM4SF1 (transmembrane 4 L6 family member 1), PGD (6-phosphogluconate dehydrogenase), G6PD (glucose-6-phosphate 1-dehydrogenase), and GSTP1 (glutathione S-transferase P), potentially enhancing glutathione biosynthesis (Figure 5C). The GSSG/GSH Quantification Kit assay showed that CGA increased GSH biosynthesis in hepatocytes after 96 h (*p* < 0.01), but did not affect the GSH/GSSG ratio (Figures 5D,E). Nevertheless, after 6 days of incubation, CGA significantly decreased the intracellular GSH/GSSG ratio (*p* < 0.001) (Figures 5D,E).

The live cell counting experiment demonstrated that co-incubation of the ferroptosis inhibitor Ferrostatin-1 (1 μM) with the IC50 concentration of CGA significantly augmented the inhibitory effect of CGA on hepatocyte proliferation compared to the control group. Conversely, co-incubation with the ferroptosis inducer Erastin (1 μM) markedly attenuated the inhibitory impact of the IC50 concentration of CGA on hepatocyte growth (Supplementary Figure S2C). Western blot analysis showed up-regulation of IREB2 (Iron-responsive element-binding protein 2) and down-regulation of Ferritin and c-Myc in hepatocytes treated with CGA at its IC50 concentration (Figure 5F), indicating iron deficiency. Subsequent proteomics and RT-qPCR analyses indicated that the IC50 of CGA may upregulate ALDH1B1 (mitochondrial aldehyde dehydrogenase X) and ACADVL (very long-chain specific acyl-CoA dehydrogenase) expression (*p* < 0.05) (Supplementary Figure S2A,B), which prevents lipid peroxidation and enhancing fatty acid degradation in hepatocytes.

### 3.5 The IC50 of CGA decreases the levels of bioavailable iron in hepatocytes, leading to disruptions in energy metabolism, redox balance, and cell cycle arrest

**FIGURE 6:**
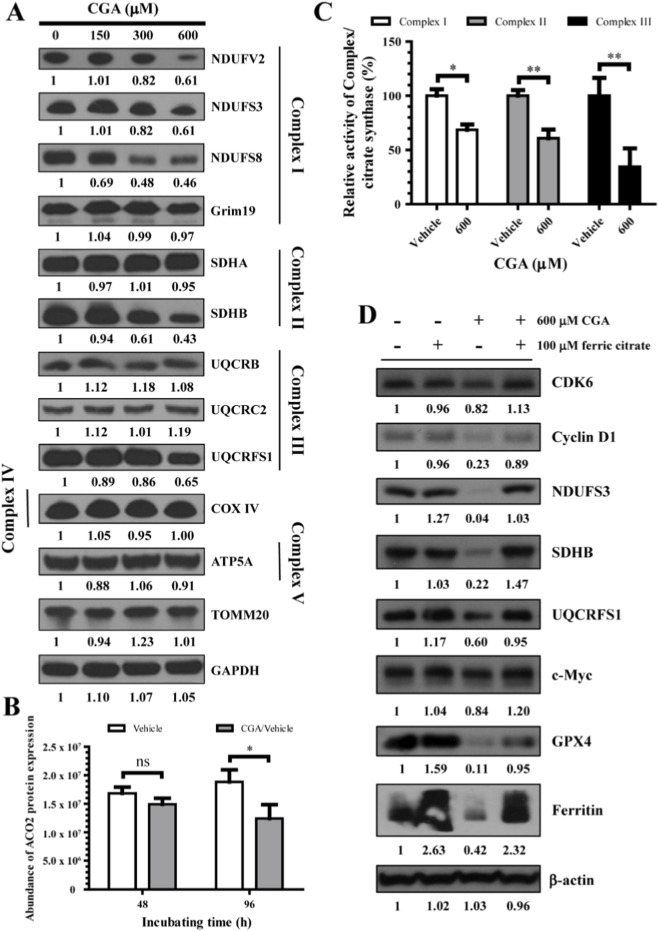
Iron deficiency due to CGA’s IC50 specifically lowers the expression of iron-sulfur cluster subunits in mitochondrial complexes I-III, hindering their activity and significantly impairing cellular energy metabolism and hepatocyte growth. **(A)** The impact of CGA on the expression of mitochondrial complex subunits encoded by nuclear genes was assessed using Western blot analysis (NDUFV2, 27 kDa; NDUFS3, 30 kDa; NDUFS8, 23 kDa; Grim19, 17 kDa; SDHA, 72 kDa; SDHB, 32 kDa; UQCRB, 14 kDa; UQCRC2, 48 kDa; UQCRFS1, 30 kDa; COX IV, 17 kDa; ATP5A, 60 kDa; TOMM20, 16 kDa). **(B)** Cis-aconitase ACO2 levels in L-02 cell mitochondria were measured after treatment with 600 μM CGA for 48 and 96 h using mass spectrometry. T-test showed significance at *p* < 0.05. **(C)** The activity of complexes I-III was observed in response to treatment with CGA, and the histogram depicted the normalized values relative to those of the untreated group. **(D)** The results of Western blot analysis indicate that the addition of 100 μM ferric citrate (exogenous Fe^3+^) to the DMEM medium containing 5% FBS resulted in a significant reduction in hepatocellular toxicity induced by CGA at a concentration of 600 μM. The data are presented as the means ± standard deviation (n = 3). ns, not significant; *p < 0.05, **p < 0.01.

The IC50 of CGA can reduce oxidative stress in hepatocytes by affecting the redox system; however, it may also deplete ferrous ions and impact various biological processes. The results shown in Figure 6 indicate a significant decrease in the protein expression levels of the iron-sulfur (Fe-S) cluster subunits (NDUFV2, NDUFS3, NDUFS8, SDHB, and UQCRFS1) in mitochondrial complexes I-III (Figure 6A), as well as in the protein expression of mitochondrial cis-aconitase ACO2 (Figure 6B), which contains an iron-sulfur cluster. This decrease leads to reduced enzyme activity in mitochondrial complexes I-III (Figure 6C). Other subunits in mitochondrial complex I-V that do not contain iron-sulfur clusters were not affected (Figure 6A). Despite the decrease in enzyme activity of mitochondrial complexes I-III, the IC50 of CGA did not significantly increase intracellular total ROS and mitochondrial ROS levels (refer to Supplementary Figure S3A,B).

To directly test the hypothesis that CGA toxicity stems from functional iron deficiency, we performed rescue experiments with ferric citrate. First, in cell viability assays, co-incubation with ferric citrate (75 or 150 µM) significantly alleviated the inhibitory effect of CGA (IC50) on hepatocyte activity (Supplementary Figure S3C). Crucially, control groups treated with the same concentrations of ferric citrate alone showed no significant effect on cell viability, confirming that the rescue was not due to non-specific growth promotion by iron. Second, we investigated the molecular basis of this rescue. Western blot analysis revealed that co-treatment with 100 µM ferric citrate effectively reversed the CGA-induced downregulation of key proteins involved in the cell cycle (CDK6, Cyclin D1, c-Myc), mitochondrial electron transport (NDUFS3, SDHB), and iron storage (Ferritin), while also modulating redox balance markers (GPX4) (Figure 6D). Importantly, the control group treated with 100 µM ferric citrate alone provided critical mechanistic insight: it did not reproduce the CGA-induced pattern of protein suppression. Instead, it led to an expected upregulation of Ferritin and had no significant effect on the levels of CDK6, Cyclin D1, c-Myc, NDUFS3, or SDHB (Figure 6D). This stark contrast demonstrates that ferric citrate specifically corrects the molecular perturbations caused by CGA rather than exerting independent, generalized effects. Additionally, to probe the specificity of the cell death pathway, co-incubation with the necrosis inhibitor Nec-1s (10 µM) partially improved cell viability, whereas the NF-κB inhibitor CAPE (1 µM) had no effect (Supplementary Figure S3D). Collectively, these findings provide multi-layered evidence that depletion of the labile iron pool is a central event in CGA-induced hepatotoxicity, and that its consequences—ranging from cell cycle arrest and mitochondrial dysfunction to altered protein expression—can be specifically counteracted by iron repletion.

### 3.6 CGA overdosage induces hepatotoxicity through the ROS signaling pathway

**FIGURE 7:**
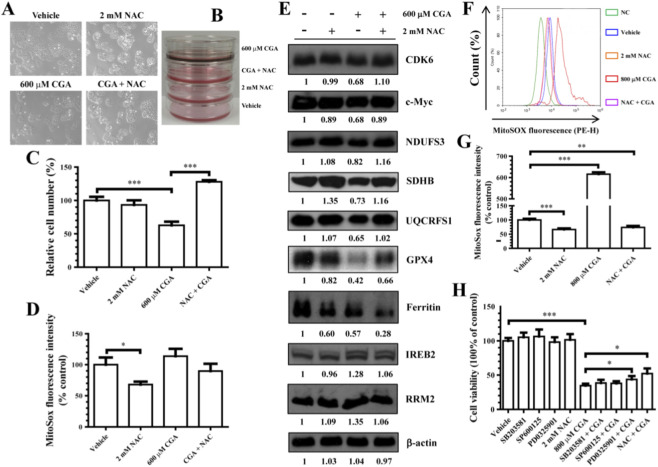
The induction of hepatotoxicity by excessive CGA is mediated through the ROS signaling pathway. **(A)** The alteration in cell morphology was noted following exposure to 600 µM CGA for a duration of 96 h, with or without the addition of 2 mM NAC. **(B)** A photograph depicting the DMEM (5% FBS) medium following a 48-h incubation of L-02 cells treated with CGA and/or NAC. **(C)** The impact of the antioxidant N-acetylcysteine (NAC) at a concentration of 2 mM on the cytotoxicity of L-02 cells induced by CGA at a concentration of 600 μM was assessed, and the number of surviving cells was quantified using flow cytometry. **(D,F,G)** MitoSOX-sensitive mitochondrial reactive oxygen species (ROS) levels were assessed via flow cytometry in L-02 cells treated with CGA at IC50 (600 μM) **(D)** or an overdose (800 μM) **(F,G)**, with or without pretreatment with NAC (2 mM). Quantitative analysis of MitoSox fluorescence intensity, normalized to untreated cells, is presented in panels **(D)** and **(G)**. **(E)** Western blot analysis was employed to assess the impact of NAC on the expression levels of key target proteins and associated signaling pathway proteins in CGA-treated L-02 cells. **(H)** L-02 cells were exposed to an excessive concentration of CGA (800 μM) in the presence or absence of a small molecule inhibitor (SB203581, 200 nM; SP600125, 200 nM; PD0325901, 20 nM; NAC, 2 mM) for a duration of 96 h, followed by assessment of cell viability using the MTT reagent. The data is reported as means ± standard deviation (n = 3). *p < 0.05, **p < 0.01, ***p < 0.001.

As previously mentioned, extended exposure of hepatocytes to the IC50 of CGA still disrupts cellular redox homeostasis. Consequently, this study investigated how the antioxidant NAC can protect hepatocytes from toxicity caused by excessive CGA. *In vitro* experiments showed that co-treatment with NAC (2 mM) and CGA (600 µM) prevented the non-enzymatic discoloration reaction of CGA and effectively mitigated the inhibitory effects of CGA on hepatocyte proliferation (Figures 7A–C). Flow cytometry analysis indicated that NAC prevented ROS accumulation in hepatocyte mitochondria caused by the IC50 of CGA (*p* > 0.05) (Figure 7D). RT-qPCR and WB experiments demonstrated that, in addition to ferritin, NAC also helped mitigate the effects of CGA on cellular processes in L-02 cells (Figure 7E; Supplementary Figure S3F).

Previous studies have shown that deferoxamine (DFO) inhibits the growth of esophageal squamous cell carcinoma (ESCC) cells by activating the ERK pathway in a ROS-dependent manner (Lan et al., 2018). This research found that CGA concentrations above 300 µM increased Erk1/2 phosphorylation (Supplementary Figure S3E), and treatment with 800 µM CGA led to a significant increase in ROS accumulation in hepatocytes (Figures 7F,G). NAC reduced mitochondrial ROS levels caused by 800 µM CGA and improved L-02 cell activity (Figures 7G,H). PD0325901 (a MEK kinase inhibitor) was also effective in mitigating the effects of CGA (800 µM) (*p* < 0.05), while SB203581 (a p38 MAPK inhibitor) and SP600125 (a broad-spectrum JNK inhibitor) were not (Figure 7H). Consequently, these experimental outcomes are consistent with a model in which the ROS-MAPK/Erk1/2 pathway may play a crucial role in mediating the hepatocyte toxicity induced by excessive CGA.

### 3.7 The LD_50_ value of CGA and its corresponding safe dose range were established through an acute toxicity test

**FIGURE 8:**
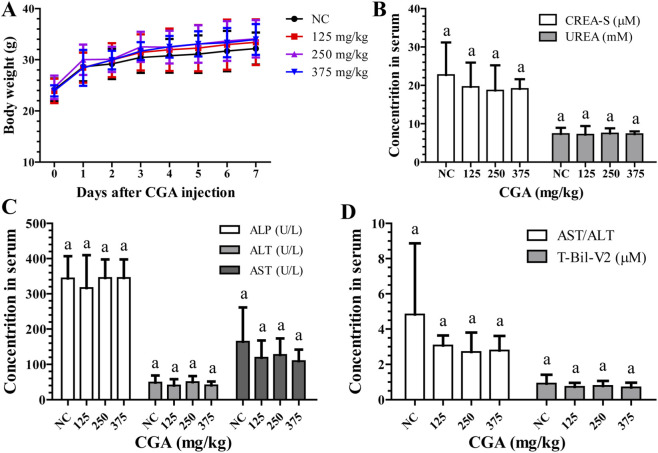
Assessment of the safety profile of CGA through *in vivo* acute toxicity testing. **(A)** Body weight was measured on days 0–7 following administration of CGA injection at doses of 0, 125, 250, or 375 mg/kg, with a sample size of 10 individuals in each group. **(B–D)** Liver and kidney function showed no significant abnormalities following treatment with CGA, as indicated by the levels of serum creatinine (CREA-S), urea nitrogen (UREA), alkaline phosphatase (ALP), alanine aminotransferase (ALT), aspartate transaminase (AST), and total bilirubin (T-Bil-V2). The control group received saline (NC). Data is presented as mean ± standard deviation. ^a–c^ Mean values not sharing a common superscript were significantly different among the groups (*p* < 0.05).

In Table 1, it is shown that all KM mice survived for 7 days after receiving 0–125 mg/kg CGA injections; however, mortality rates increased with higher doses, reaching 100% at 500 mg/kg. Symptoms such as convulsions and difficulty breathing were observed in mice receiving higher doses. Nevertheless, mice injected with different doses of CGA did not show significant changes in body weight or serum biochemical indices related to liver and kidney function compared to the control group over a 7-day period (Figures 8A–D). Based on the data, the LD50 of CGA is 382.28 mg/kg, indicating a safe dosage range for a single intravenous injection of 0–125 mg/kg. Considering an estimated average blood volume of approximately 7%–8% of body weight in mice (Diehl et al., 2001), the LD50 dose of 382.28 mg/kg would result in an initial circulating concentration estimated to be in the range of 5–10 mM. This concentration is approximately an order of magnitude higher than the *in vitro* IC50 of 613 µM for hepatocytes, taking into account the rapid distribution and metabolism occurring *in vivo*.

**TABLE 1:** The LD50 test results were determined through the intravenous injection of CGA into the tail vein (n = 10).

Groups	Mouse (number)	Dosage (mg/kg)	Number of deaths	Death rate (%)	LD50, 95% confidence limit, average confidence limit
1	10	0	0	0	382.28 mg/kg, 332.11 ∼ 441.98 mg/kg, 382.28 ± 54.94 mg/kg
2	10	125	0	0	
3	10	250	1	10%	
4	10	375	3	30%	
5	10	500	10	100%	

## 4 Discussion

Research indicates that CGA is generally safe for humans, with complex absorption and metabolism involving the gastrointestinal tract and liver (Salomone et al., 2017; Monteiro et al., 2007). This safety is reflected in a substantial body of literature documenting its hepatoprotective effects against various insults, such as damage induced by cyclophosphamide (Hao et al., 2024), carbon tetrachloride (CCl_4_)-induced fibrosis (Ne et al., 2025), and acetaminophen (APAP) overdose (Yao et al., 2025; Wei et al., 2025). This protective effect is mediated through mechanisms such as the enhancement of antioxidant enzymes, including superoxide dismutase (SOD) and GSH, activation of the Nrf2 pathway, and modulation of autophagy via the PI3K/AKT/mTOR and AMPK/mTOR/ULK1 pathways. Additionally, in avian models, specifically laying hens, CGA has been shown to enhance liver health through the gut-liver axis by attenuating inflammation and oxidative stress (Hu et al., 2025). These findings are consistent with our observations that lower concentrations of CGA (50–200 µM) protect hepatocytes and enhance mitochondrial energy metabolism *in vitro*. The safety of CGA is further supported by *in vivo* findings; a single intravenous dose of up to 125 mg/kg was well-tolerated in mice in our study, aligning with reports of safe intraperitoneal administration at even higher doses (Huang et al., 2019). A recent comprehensive review affirms CGA’s general safety profile across a wide range of applications and doses (Nguyen et al., 2024).

**FIGURE 9:**
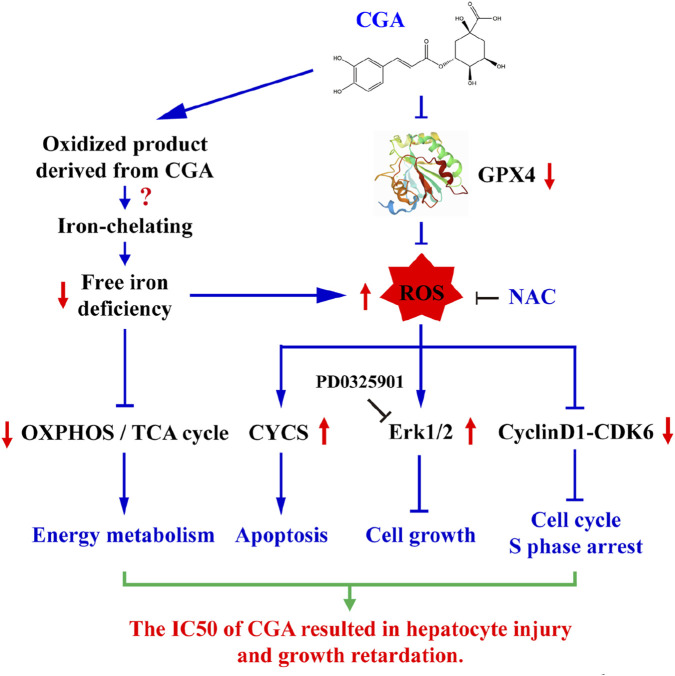
A schematic diagram is presented to elucidate the molecular mechanism underlying hepatocyte growth arrest and injury induced by CGA at its IC50 concentration.

However, emerging evidence and the findings of this study reveal a critical, context-dependent duality in CGA’s action. While safe and beneficial in common dietary or therapeutic contexts, excessive or direct high-concentration exposure can be detrimental (Amigo-Benavent et al., 2017; Wang et al., 2019). Our work demonstrates that CGA concentrations exceeding 600 µM *in vitro* significantly inhibit hepatocyte growth, clonogenicity, and viability, and induce acute toxicity at high intravenous doses *in vivo*. This apparent paradox is resolved by considering the specific exposure scenario. In models of pre-existing oxidative stress or injury, CGA’s potent antioxidant properties are therapeutic (Shi et al., 2021; Ji et al., 2024). In contrast, our experimental model investigates the direct effect of supra-physiological, unmetabolized CGA on otherwise normal hepatocytes—a scenario relevant to overdose or the use of highly concentrated preparations. Under these specific conditions, we identify a novel toxic mechanism primarily driven by CGA’s ability to chelate iron, leading to a state of functional iron deficiency (Figure 9).

The core of this toxicity mechanism lies in the depletion of the labile Fe^2+^ pool (Figure 5B)—without altering total cellular iron (Figure 5A)—which triggers a compensatory iron-starvation response (IREB2↑, TfR1↑, Ferritin↓; Figures 5F; Supplementary Figure S2A,B). This functional iron deficiency has a profound and specific downstream consequence: the impaired synthesis or stability of mitochondrial Fe-S cluster proteins. We observed a significant decrease in the expression of Fe-S subunits in respiratory complexes I-III and the Fe-S enzyme aconitase 2 (ACO2), leading directly to reduced electron transport chain activity (Figures 6A–C). This specific impairment of mitochondrial energy metabolism, combined with the downregulation of key cell cycle regulators such as c-Myc and Cyclin D1, provides a direct mechanistic explanation for the observed S-phase arrest and growth inhibition. The causality of this iron-deficiency pathway is robustly supported by rescue experiments; the toxicity was specifically reversed by co-treatment with ferric citrate, while ferric citrate alone did not reproduce the toxic effects (Figure 6D; Supplementary Figure S3C; Figure 9).

The interaction between CGA-induced iron deficiency and regulated cell death pathways is complex and context-dependent. The cells initially mounted adaptive responses, upregulating enzymes involved in glutathione biosynthesis (RRM2, GSR, GCLM) and fatty acid degradation (ALDH1B1, ACADVL). Notably, the ferroptosis inhibitor Ferrostatin-1 exacerbated CGA’s toxicity, while the inducer Erastin attenuated it (Supplementary Figure S2C), suggesting that the profound iron deficiency we describe disrupts fundamental metabolism in a manner distinct from, and prior to, the execution of classical ferroptosis—a pathway CGA has been shown to inhibit in other contexts via Nrf2/GPX4 activation (Yang et al., 2025). Nevertheless, with prolonged exposure or at higher concentrations, the homeostatic balance is overwhelmed. The GSH/GSSG ratio decreased after extended incubation (Figures 5D,E), and a concentration of 800 µM CGA induced significant ROS accumulation, activating the ROS-Erk1/2 stress pathway (Figure 7; Supplementary Figure S3E), indicating that oxidative stress contributes to the toxicity under conditions of severe overdose (Figure 9).

The mechanistic insights from this study have implications beyond explaining toxicity. They provide a scientific basis for the rational application of CGA’s iron-chelating property, such as in the design of functional materials to modulate iron absorption (Zhang et al., 2024) or in forming bioactive complexes with proteins like lactoferrin (Li et al., 2024). The biological outcome—protection or toxicity—is critically determined by the concentration of CGA and its relative availability to cellular iron pools. Future research should explore whether different CGA isomers, known to vary in antioxidant potency (Cheng et al., 2025), also differ in their iron-chelating capacity and toxicological profile. Furthermore, investigating CGA’s potential to chelate other pro-oxidant heavy metals following oxidation could open new avenues for therapeutic intervention.

In conclusion, this study elucidates a distinct concentration-dependent duality of CGA in hepatic cells. At low-to-moderate concentrations, CGA exhibits predominant antioxidant properties, conferring cytoprotection. Conversely, at high concentrations under direct exposure conditions, its iron-chelating properties become predominant, leading to a functional iron deficiency that specifically impairs mitochondrial Fe-S cluster proteins, resulting in an energy metabolism crisis and cell cycle arrest. By establishing a safe *in vitro* concentration range and a safe intravenous dose, alongside elucidating the precise molecular mechanisms underlying its toxicity, this research provides a critical framework for evaluating the risk-benefit balance of CGA. This framework is instrumental in guiding its safe and effective application in dietary, clinical, and industrial contexts.

## 5 Limitations

This study provides novel insights into the dual role of CGA, but several limitations should be acknowledged to guide future research.

First, while the rescue experiments with ferric citrate and NAC strongly support an iron deficiency and ROS-mediated mechanism, the absence of established positive controls, such as the iron chelator deferoxamine (DFO) or direct ROS generators, precludes a definitive comparative conclusion that the observed effects are uniquely attributable to CGA’s iron-chelating activity. Incorporating such controls in future studies would strengthen the causal inference.

Second, the chemical form of CGA responsible for toxicity warrants further investigation. It remains to be determined whether the parent compound, its oxidized form, or a specific metabolite is the primary mediator of the observed hepatotoxicity and iron chelation.

Third, regarding the *in vitro* assessment of hepatocyte injury, this study primarily relied on LDH release, apoptosis, and cell cycle arrest. Although changes in alkaline phosphatase (ALP) were noted, alanine aminotransferase (ALT)—a more specific circulating biomarker for hepatocellular necrosis—was not measured in the culture supernatants. The lack of significant serum ALT elevation in mice treated with sub-lethal CGA doses (Figure 8C) aligns with the *in vitro* findings where programmed cell death was predominant. Future *in vitro* studies should include ALT measurement for a more comprehensive cytotoxicity profile.

Fourth, specific aspects of the experimental design could be refined. Direct measurement of labile Fe^2+^ and total iron levels in cells following co-treatment with ferric citrate and CGA was not performed. Such data would provide more direct evidence for the chelation-rescue mechanism by confirming the restoration of the intracellular iron pool.

Fifth, the acute systemic toxicity mechanism in KM mice following high-dose (≥250 mg/kg) tail vein injection was not deeply explored. The rapid mortality suggests potential acute effects on other systems (e.g., central nervous or cardiovascular) beyond the hepatocellular iron-deficiency pathway characterized *in vitro*. A more complete toxicological profile requires future investigations incorporating time-course histopathology (e.g., H&E staining of liver tissue), analysis of tissue-specific iron distribution, and measurement of systemic acute-phase response markers.

## 6 Conclusion

In conclusion, this study delineates a concentration-dependent duality in the action of CGA on hepatocytes, defined by its iron-chelating property. At low to moderate concentrations (50–200 µM), CGA exerts protective effects, likely mediated by its antioxidant capacity. In contrast, at high concentrations (≥600 µM *in vitro*), CGA induces a state of functional iron deficiency by depleting the labile Fe^2+^ pool. This deficiency specifically impairs mitochondrial Fe-S cluster proteins, leading to disruptions in electron transport chain activity, cellular energy metabolism, and cell cycle progression, ultimately resulting in growth inhibition and apoptosis. *In vivo*, a single intravenous dose of up to 125 mg/kg was found to be safe in mice, with an LD50 of 382.28 mg/kg. These findings establish a mechanistic link between excessive CGA exposure, cellular iron deprivation, and mitochondrial dysfunction, providing a crucial scientific basis for defining its safe dosage window and understanding its potential hepatotoxic risk under conditions of overdose or highly concentrated exposure.

## Data Availability

The mass spectrometry proteomics data have been deposited to the ProteomeXchange Consortium (https://proteomecentral.proteomexchange.org) via the iProX partner repository (J. Ma et al., 2019; Chen et al., 2022) with the dataset identifier PXD051998.

## Glossary

CGA: Chlorogenic acid
IC50: half-maximal inhibitory concentration
WB: Western blotting
RT-qPCR: real-time quantitative polymerase chain reaction
ROS: reactive oxygen species
NAC: N-Acetyl-L-cysteine
Nrf2: nuclear factor erythroid 2-related factor 2
**LD**_**50**_: semi-lethal concentration
CQA: caffeoylquinic acid
CA: caffeic acid
QA: quinic acid
DPPH: 1,1-diphenyl-2-picrylhydrazyl radical
ANIT: alpha-naphthylisothiocyanate
APAP: Acetaminophen
EGFR: Epidermal growth factor receptor
ALP: alkaline phosphatase
ALT: alanine aminotransferase
EGR1: early growth response-1
iNOS: inducible nitric oxide synthase
GO: gene ontology
KEGG: Kyoto Encyclopedia of Genes and Genomes
DMEM: Dulbecco’s Modified Eagle Medium
FBS: fetal bovine serum
CCK8: Cell Counting Kit-8
RTCA: real-time cell analysis
LDH: lactate dehydrogenase
LFQ-MS: label-free quantitative mass spectrometry
ALP: alkaline phosphatase
OCR: oxygen consumption rate
ECAR: extracellular acidification rate
FCCP: mitochondrial oxidative phosphorylation uncoupler
c-Myc: Myc proto-oncogene protein
MAPK: mitogen-activated protein kinase
Erk1/2: extracellular signal-regulated kinase 1/2
STAT3: signal transducer and activator of transcription 3
GSH: glutathione
GSSG: glutathione disulfide
PBS: phosphate buffered saline
ICP-MS: inductively coupled plasma mass spectrometry
KM mice: Kunming mice
CTSD: cathepsin D
CAPN1: calpain 1
CYCS: cytochrome c
**CCl**_**4**_: Carbon tetrachloride
EIF2S1: eukaryotic translation initiation factor 2 subunit
TfR1: transferrin receptor 1
GPX4: glutathione peroxidase 4
SLC7A11: cystine/glutamate transporter
SOD: superoxide dismutase
Chac1: glutathione-specific gamma-glutamylcyclotransferase 1
RRM2: ribonucleoside-diphosphate reductase subunit M2
GSR: glutathione reductase
GCLM: glutamate--cysteine ligase regulatory subunit
TM4SF1: transmembrane 4 L6 family member 1
PGD: 6-phosphogluconate dehydrogenase
G6PD: glucose-6-phosphate 1-dehydrogenase
GSTP1: glutathione S-transferase P
IREB2: Iron-responsive element-binding protein 2
ALDH1B1: mitochondrial aldehyde dehydrogenase X
ACADVL: very long-chain specific acyl-CoA dehydrogenase
CAPE: Caffeic acid phenethyl ester
DFO: deferoxamine
ESCC: esophageal squamous cell carcinoma.
