# Supplementary material for: Safe dosage and potential risks of chlorogenic acid: insights from in vitro and in vivo studies
Source: Front Pharmacol. 2026 Feb 24;17:1740609. doi: 10.3389/fphar.2026.1740609 (PMC12972752; doi:10.3389/fphar.2026.1740609)
Supplement: Supplementary file 1 [file Supplementaryfile1.docx]

Supplementary Material

##
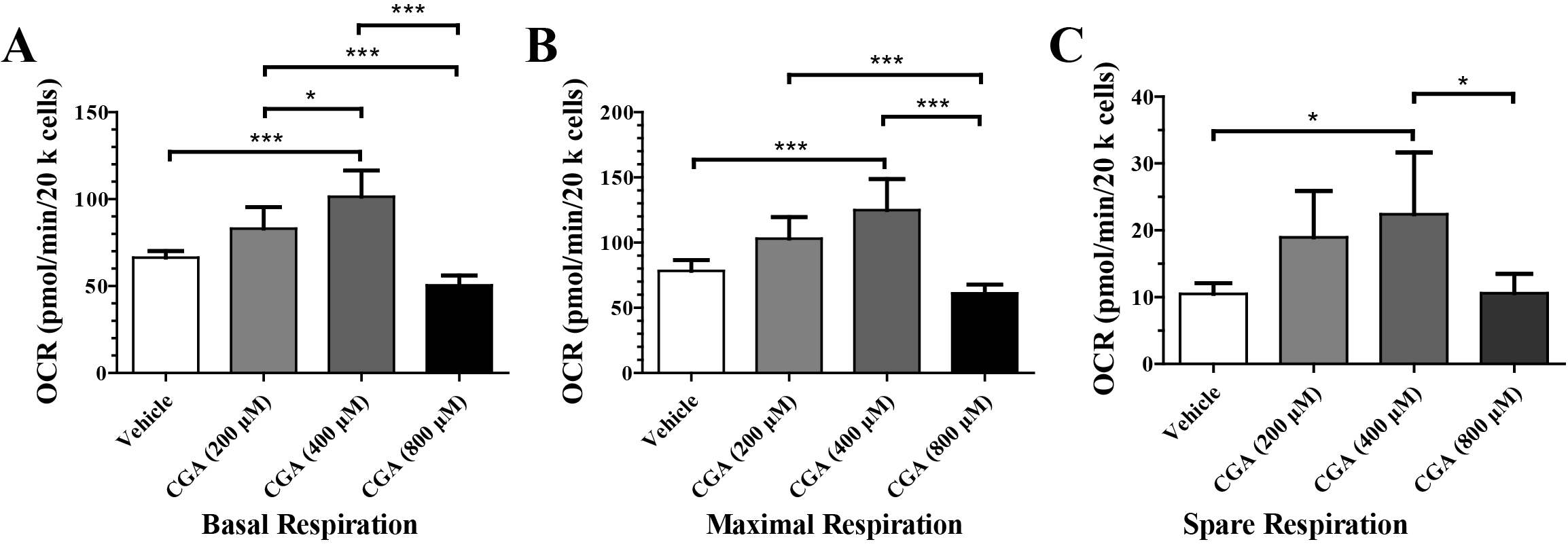


**Supplementary Figure S1**

Adequate levels of CGA enhance the process of oxygen consumption rate (OCR) in hepatocytes. **(A)** Basal OCR. **(B)** Maximal OCR. **(C)** Spare OCR. The data are presented as the mean ± standard deviation (n = 8). **p* < 0.05, ****p* < 0.001.


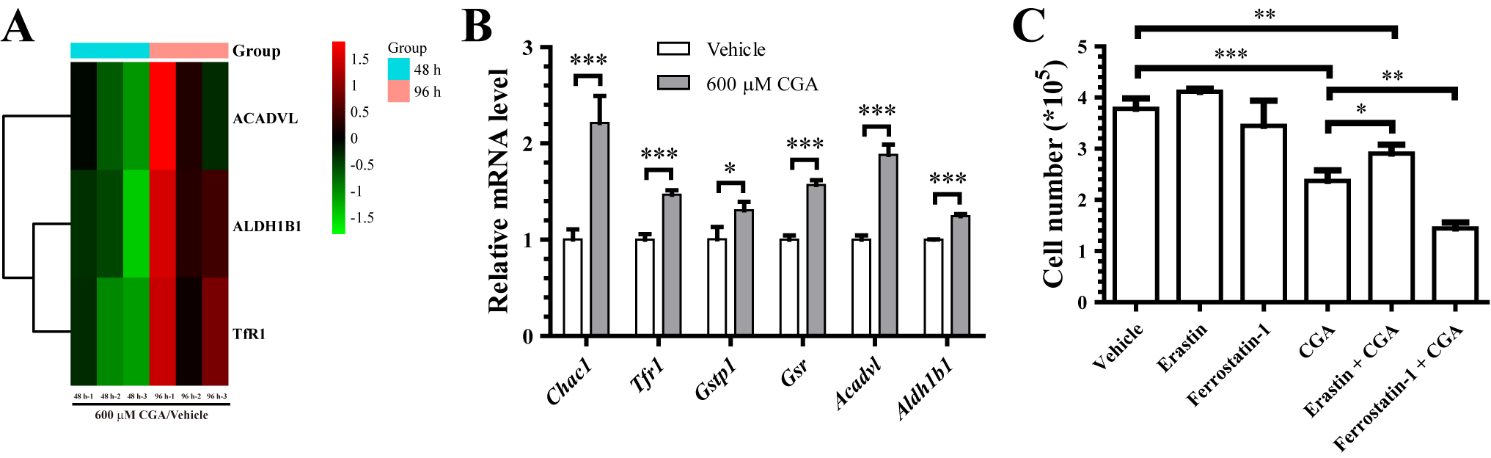


**Supplementary Figure S2**

The IC50 of CGA plays a critical role in determining the fate of hepatocytes through its dual regulation of ferroptosis homeostasis. **(A)** A heat map and dendrogram were utilized to illustrate the differential expression of fatty acid degradation and ferroptosis-related proteins at two distinct time points following treatment with CGA. **(B)** The impact of CGA on the expression levels of marker genes associated with ferroptosis, glutathione metabolism, and fatty acid degradation in L-02 cells was assessed using RT-qPCR. Target gene expression levels were normalized to β-actin and analyzed via the 2^-ΔΔCT^ method. **(C)** L-02 cells were treated with 600 μM CGA for 96 h with or without ferroptosis inducer Erastin (1 μM) and ferroptosis inhibitor Ferrostatin-1 (1 μM), and the surviving cells were counted using flow cytometry. Data is shown as mean ± standard deviation (n = 3). **p* < 0.05, ***p* < 0.01, ****p* < 0.001.


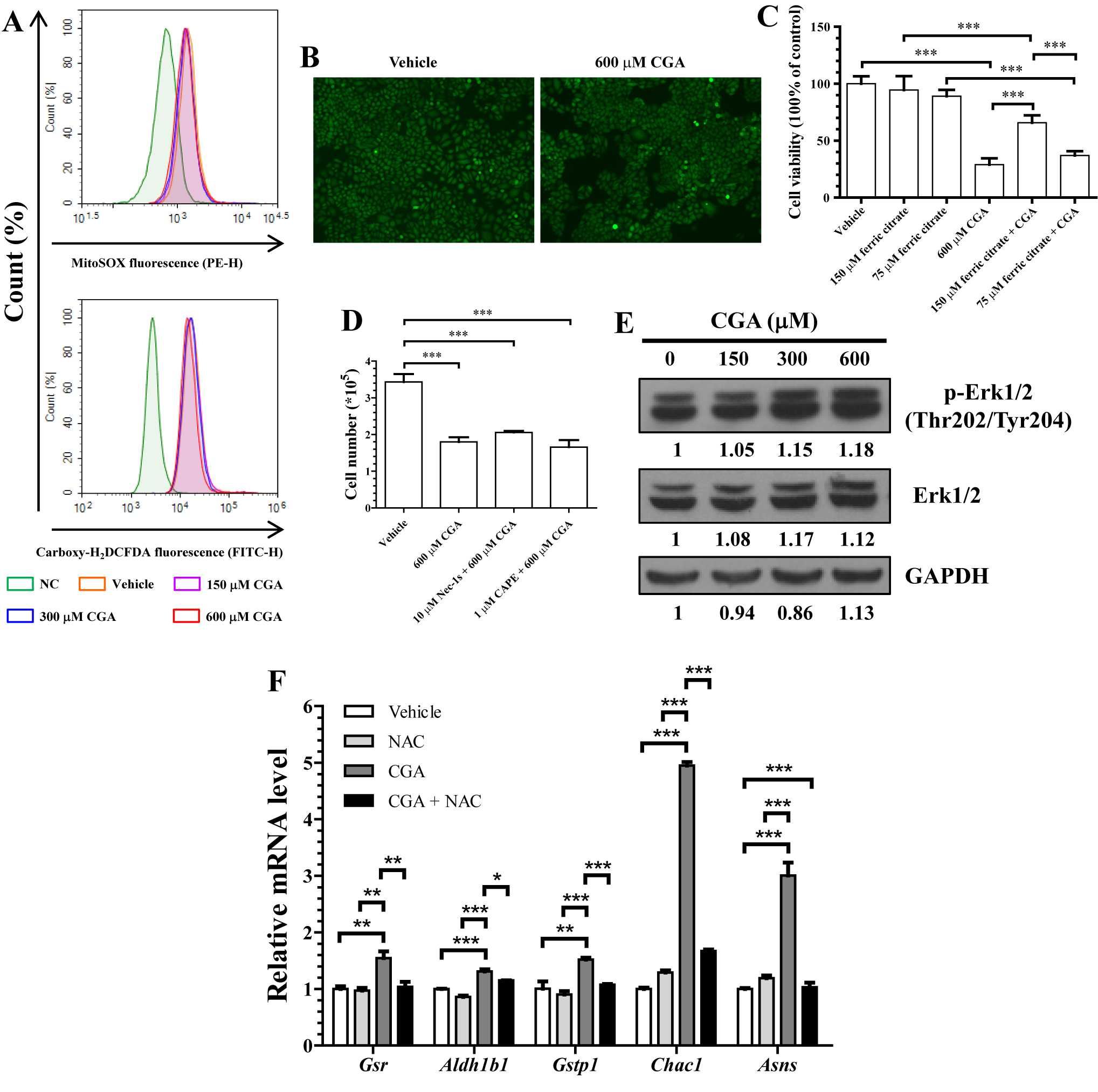


**Supplementary Figure S3**

The primary molecular mechanisms underlying the hepatocyte toxicity of the IC50 of CGA involve iron deficiency and the activation of the ROS signaling pathway. **(A)** Flow cytometry was performed to measure the mitochondrial and cellular ROS by the MitoSOX and Carboxy-H_2_DCFDA probe in L-02 cells with or without gradient concentration of CGA treated for 96 h. **(B)** Representative images of Carboxy-H_2_DCFDA probe staining in CGA treated L-02 cells which observed by fluorescence microscope. **(C)** Hepatocytes were exposed to the IC50 of CGA for a duration of 96 h, with or without varying concentrations of ferric citrate. Subsequently, cell viability was evaluated using the MTT reagent. **(D)** The effects of Nec-1s, a necrosis inhibitor, and CAPE, a specific NF-κB-activatable inhibitor, on L-02 cells treated with IC50 concentration of CGA were analyzed by flow cytometry. **(E)** L-02 cells were subjected to varying concentrations of CGA for a duration of 96 h, followed by a western blot analysis to assess the levels of phospho-ERK proteins (Erk1/2, 42/44 kDa). **(F)** The impact of NAC at a concentration of 2 mM on the modulation of key gene expression in L-02 cells stimulated by CGA at a concentration of 600 μM was assessed using RT-qPCR. The data are presented as the mean ± standard deviation (n = 3). **p* < 0.05, ***p* < 0.01, ****p* < 0.001.

**Supplementary Table S1 List of primers for qRT-PCR.**

| Primer | Sequence (5′→ 3′)^a^ | Size of PCR products (bp) |
| --- | --- | --- |
| *Cyclin D1*-QPCR-F | GCAGACCTTCGTTGCCCTCT | 148 |
| *Cyclin D*1-QPCR-R | GCGTGTGAGGCGGTAGTAGG | 148 |
| *p21*-QPCR-F | GGGGATGTCCGTCAGAACCC | 103 |
| *p21*-QPCR-R | CCGCCATTAGCGCATCACAG | 103 |
| *Asns*-QPCR-F | ATTTGGGCGCTGTTTGGCAG | 150 |
| *Asns*-QPCR-R | AACTACCGCCAACCGGTGAA | 150 |
| *Chac1*-QPCR-F | AAGGGGAGCAGGTAAGCAAGG | 122 |
| *Chac1*-QPCR-R | TGCCTTCAGTGGTTGGTCAGG | 122 |
| *Tfr1*-QPCR-F | GATTCAGGTCAAAGACAGCGCTC | 130 |
| *Tfr1*-QPCR-R | CAGTTTACCAGTAACTGTTGCAGCC | 130 |
| *Gstp1*-QPCR-F | CAGGAGGGCTCACTCAAAGC | 127 |
| *Gstp1*-QPCR-R | TCCCATAGAGCCCAAGGGTG | 127 |
| *Gsr*-QPCR-F | GCCGCAGCGTCATTGTTGG | 112 |
| *Gsr*-QPCR-R | ACCTCCTTGACCTTATCATGCCG | 112 |
| *Acadvl*-QPCR-F | CGGAAGCTCGCGGCTCA | 130 |
| *Acadvl*-QPCR-R | CCTGGTCAGAGCGTCAGAGG | 130 |
| *Aldh1b1*-QPCR-F | CCTTCACCGGTTCCACCGAG | 130 |
| *Aldh1b1*-QPCR-R | TGCTCCATGTCAGCATCGGC | 130 |
| *β-actin*-QPCR-F | AGCACAGAGCCTCGCCTTTG | 120 |
| *β-actin*-QPCR-R | AAGCCGGCCTTGCACATG | 120 |
