# Supplementary material for: Safe dosage and potential risks of chlorogenic acid: insights from in vitro and in vivo studies
Source: Front Pharmacol. 2026 Feb 24;17:1740609. doi: 10.3389/fphar.2026.1740609 (PMC12972752; doi:10.3389/fphar.2026.1740609)
Supplement: Supplementary file 2 [file Supplementaryfile2.pdf]

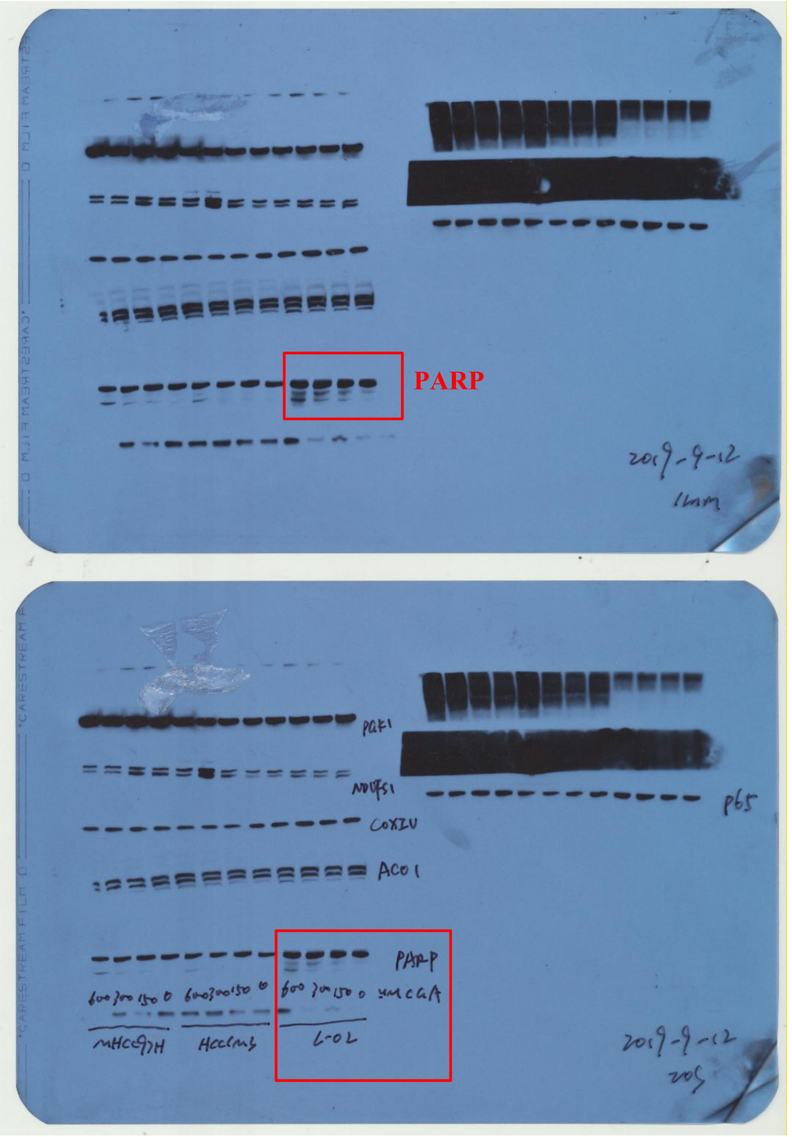

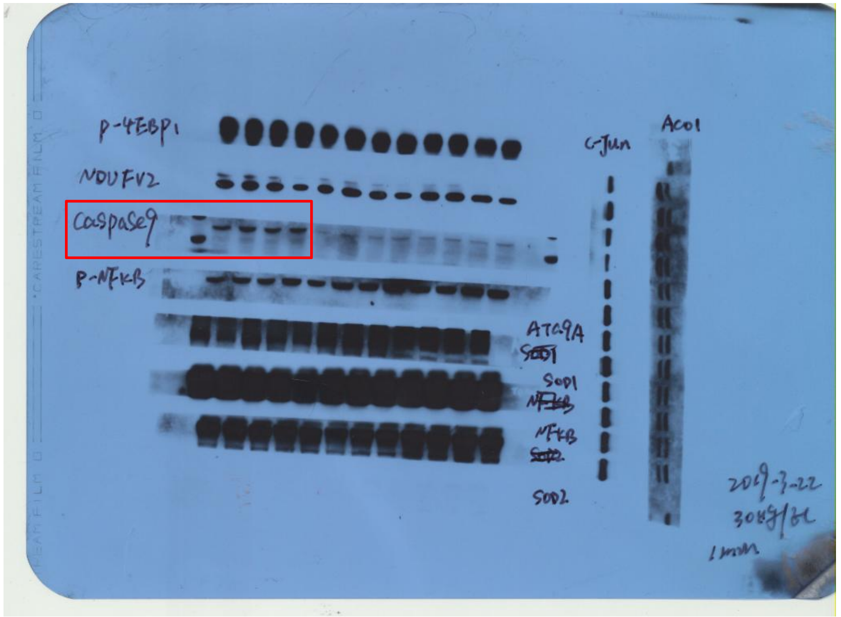

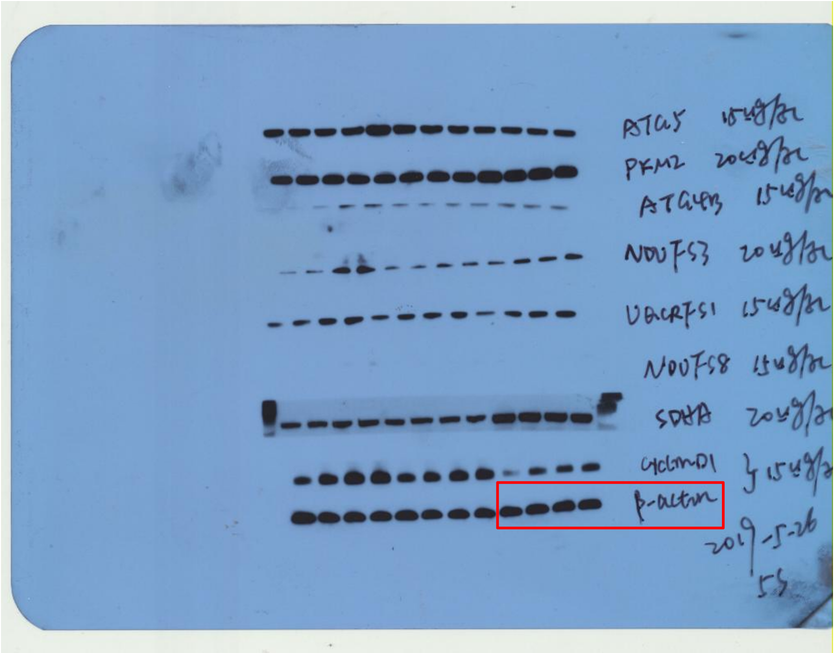

SUPPLEMENTARY FIGURE S4, related to FIGURE 4E. The raw data from the Western blotting experiment.

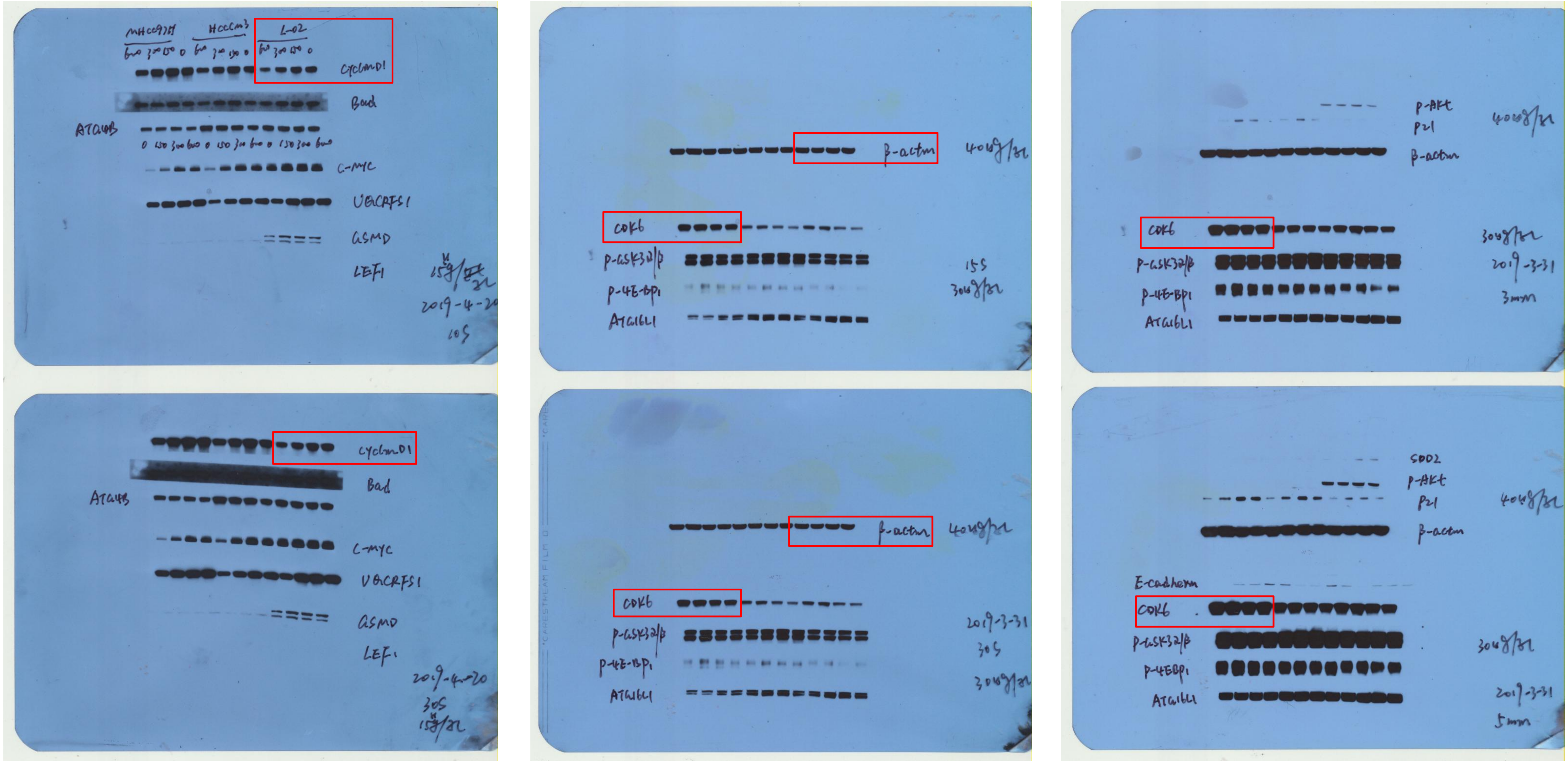

SUPPLEMENTARY FIGURE S5, related to FIGURE 4H. The raw data from the Western blotting experiment.

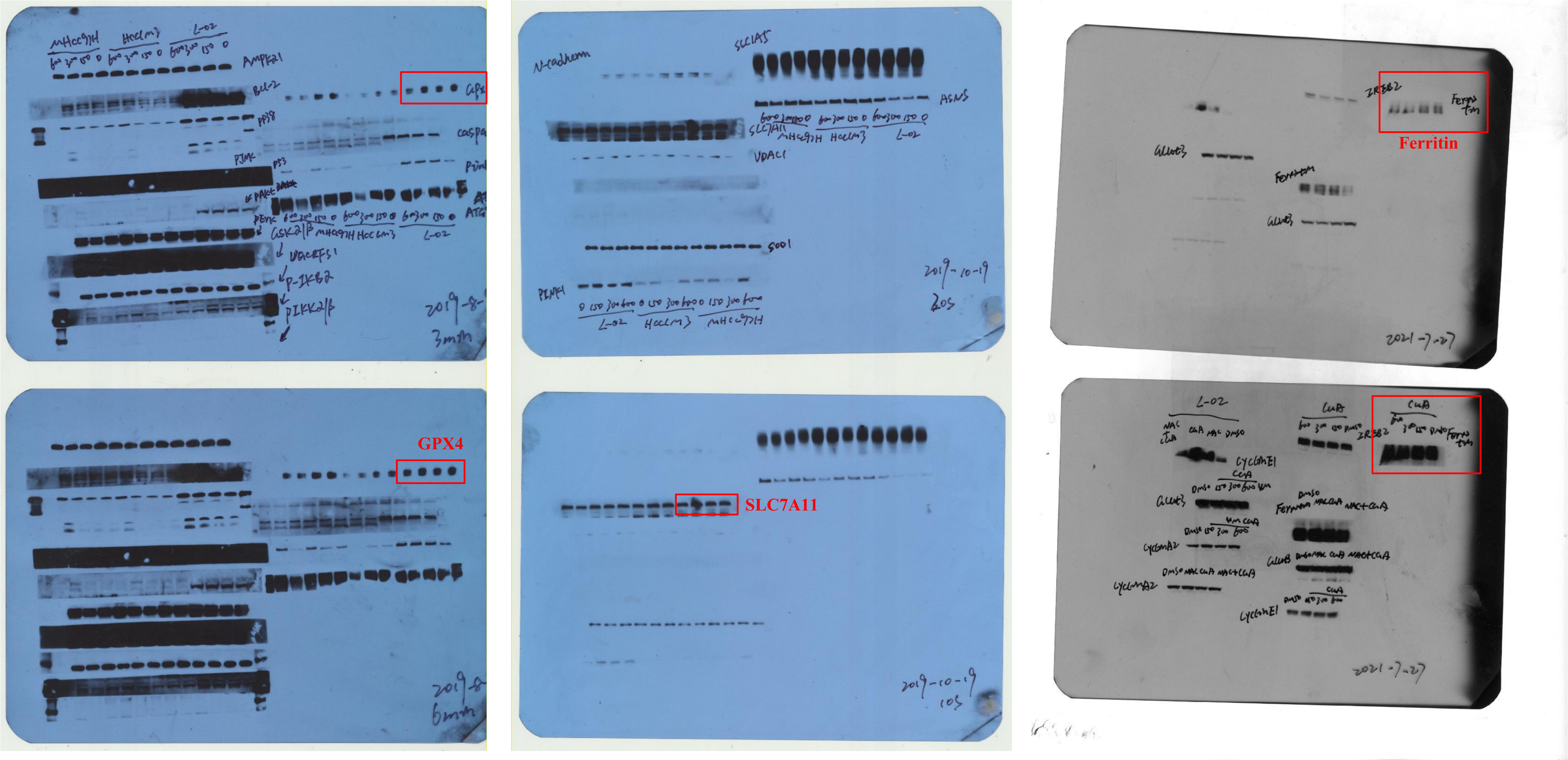

SUPPLEMENTARY FIGURE S6, related to FIGURE 5F. The raw data from the Western blotting experiment.

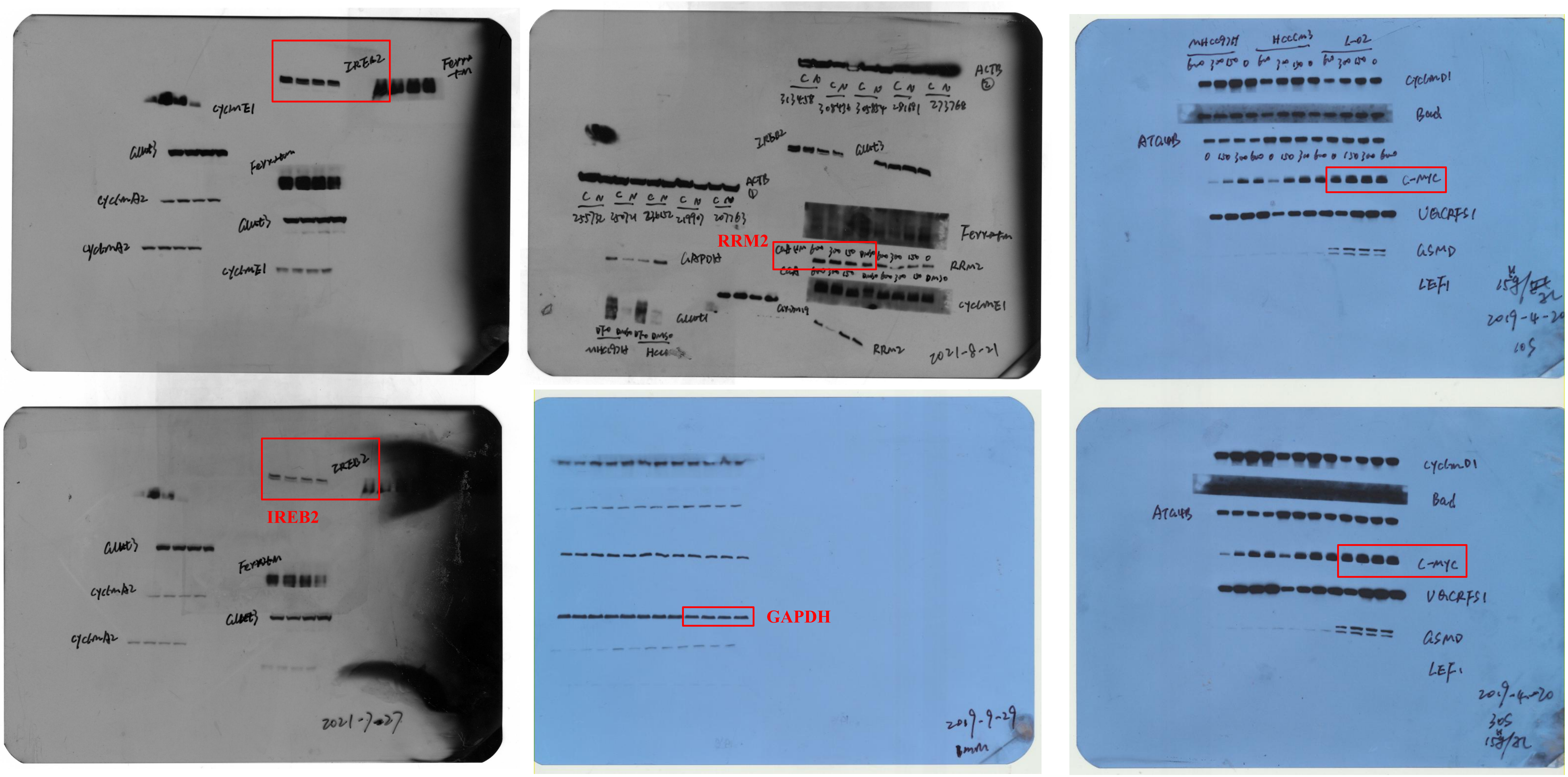

SUPPLEMENTARY FIGURE S6, related to FIGURE 5F. The raw data from the Western blotting experiment.

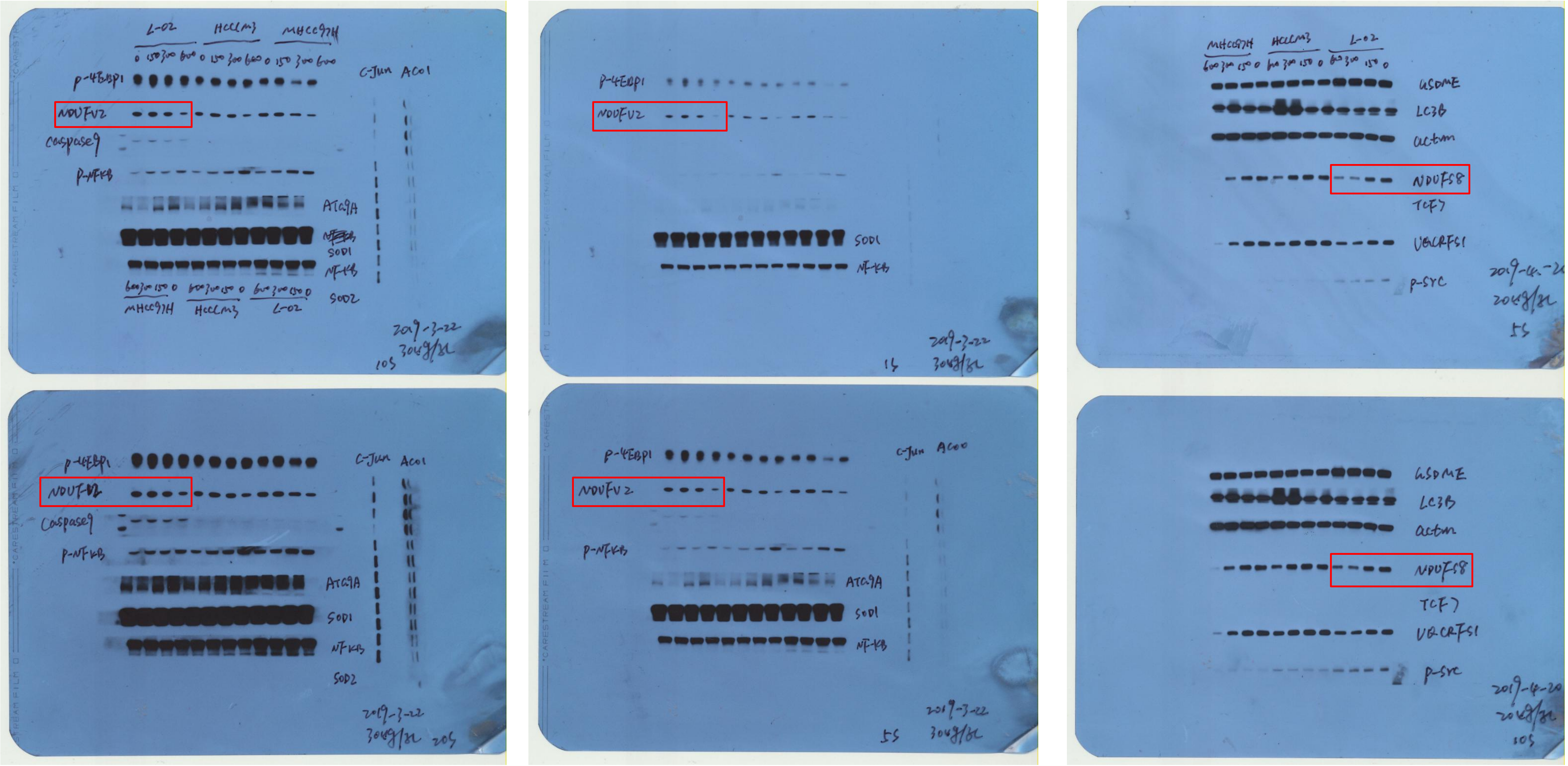

SUPPLEMENTARY FIGURE S7, related to FIGURE 6A. The raw data from the Western blotting experiment.

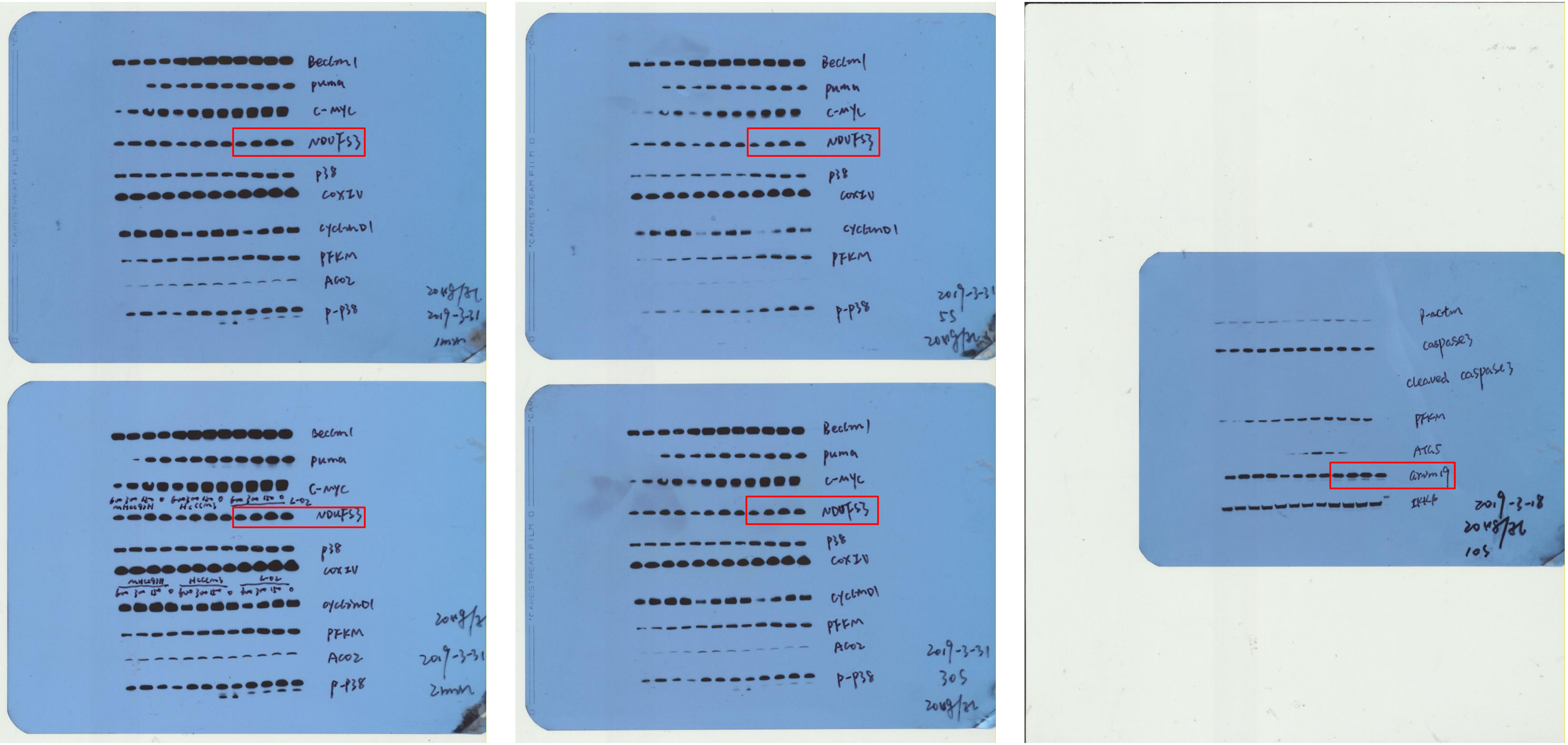

SUPPLEMENTARY FIGURE S7, related to FIGURE 6A. The raw data from the Western blotting experiment.

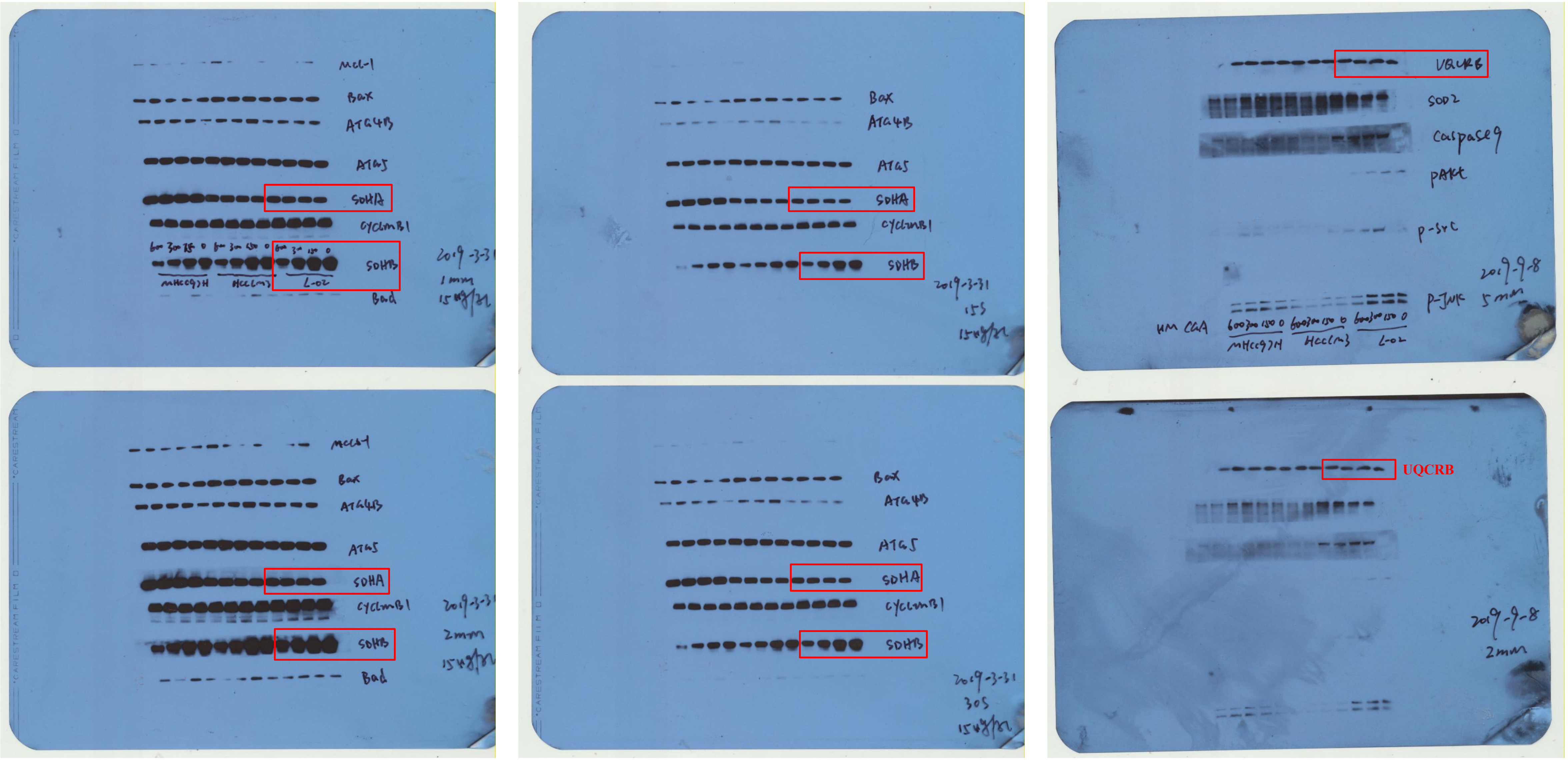

SUPPLEMENTARY FIGURE S7, related to FIGURE 6A. The raw data from the Western blotting experiment.

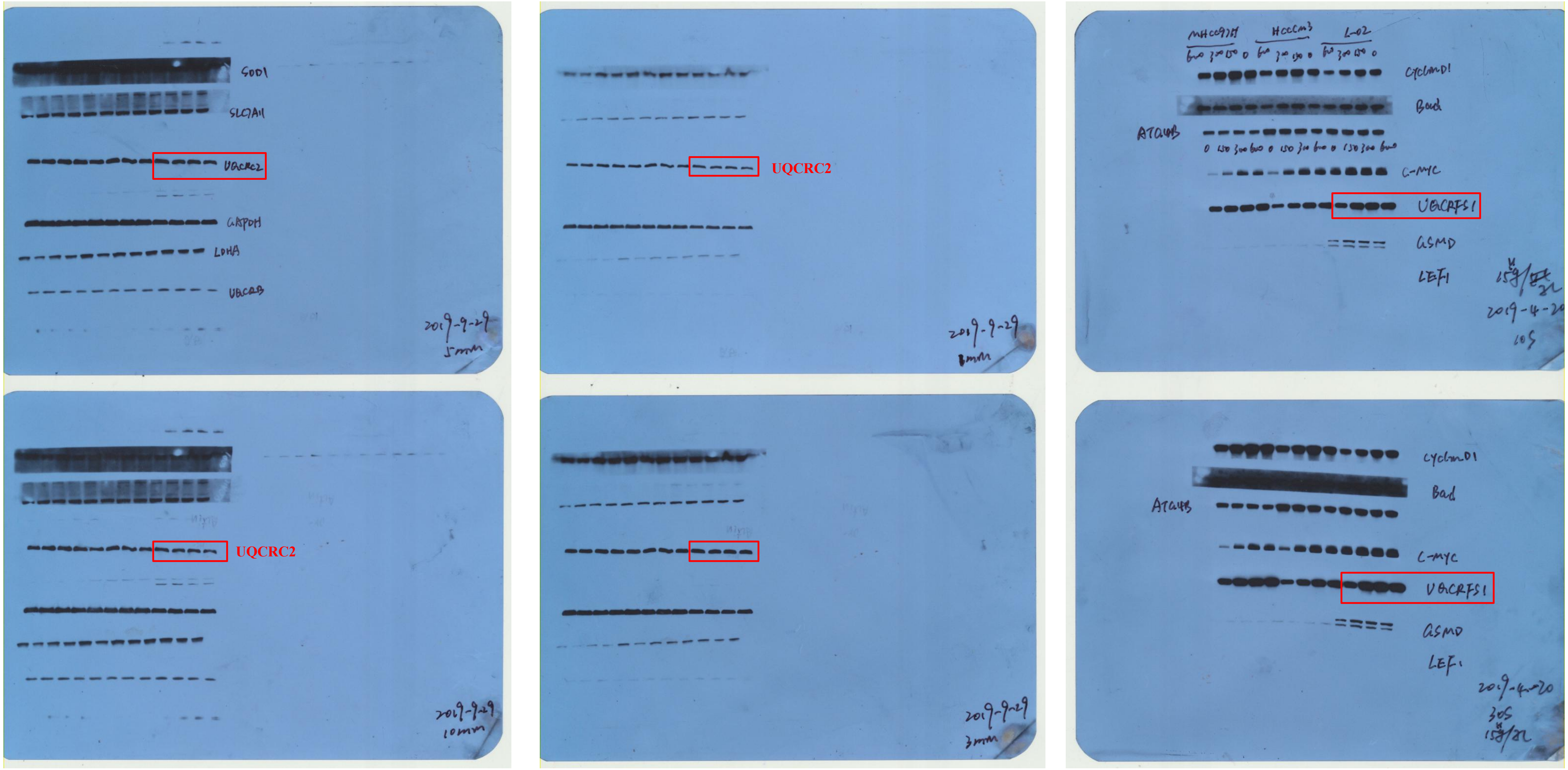

SUPPLEMENTARY FIGURE S7, related to FIGURE 6A. The raw data from the Western blotting experiment.

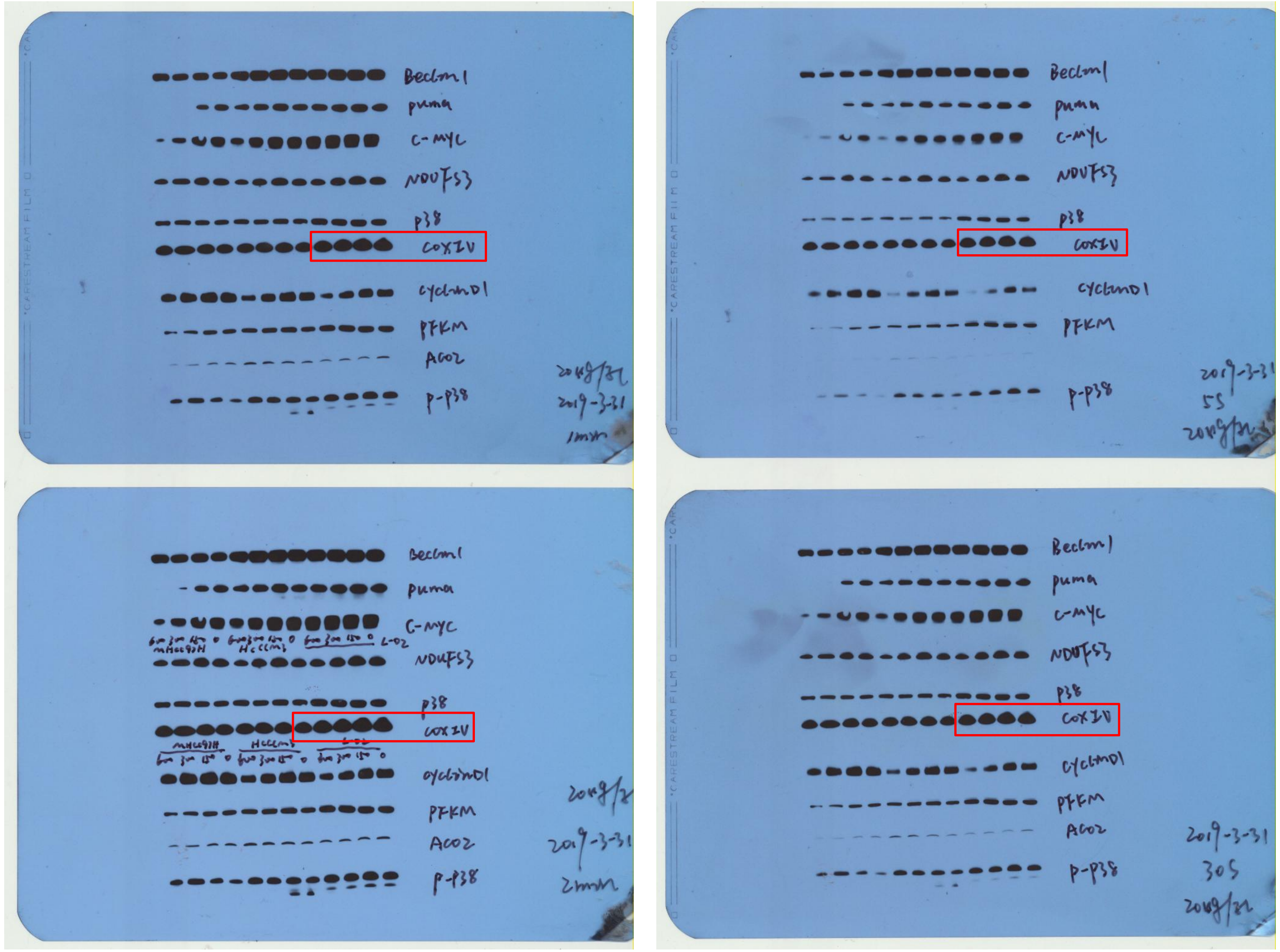

SUPPLEMENTARY FIGURE S7, related to FIGURE 6A. The raw data from the Western blotting experiment.

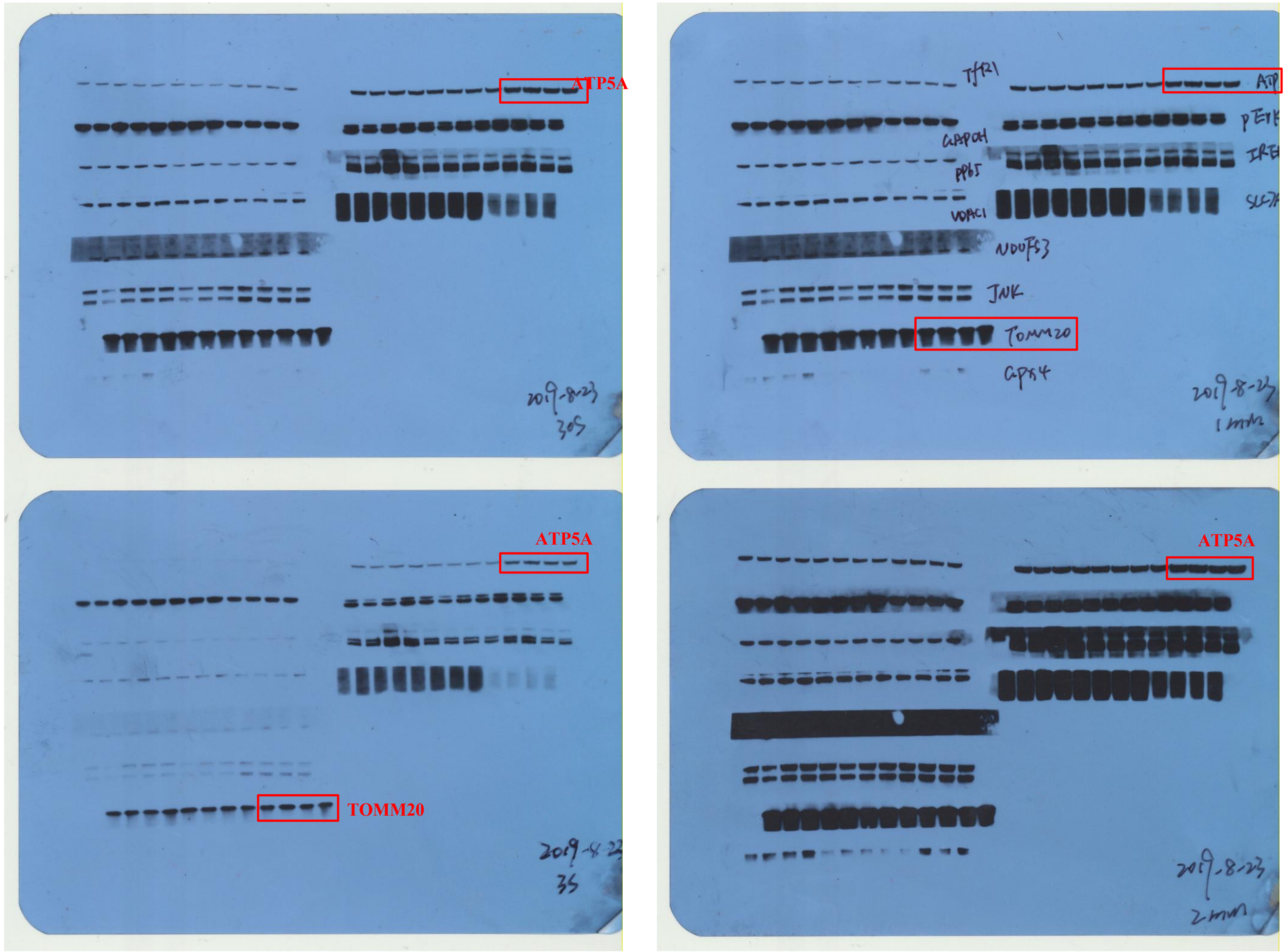

SUPPLEMENTARY FIGURE S7, related to FIGURE 6A. The raw data from the Western blotting experiment.

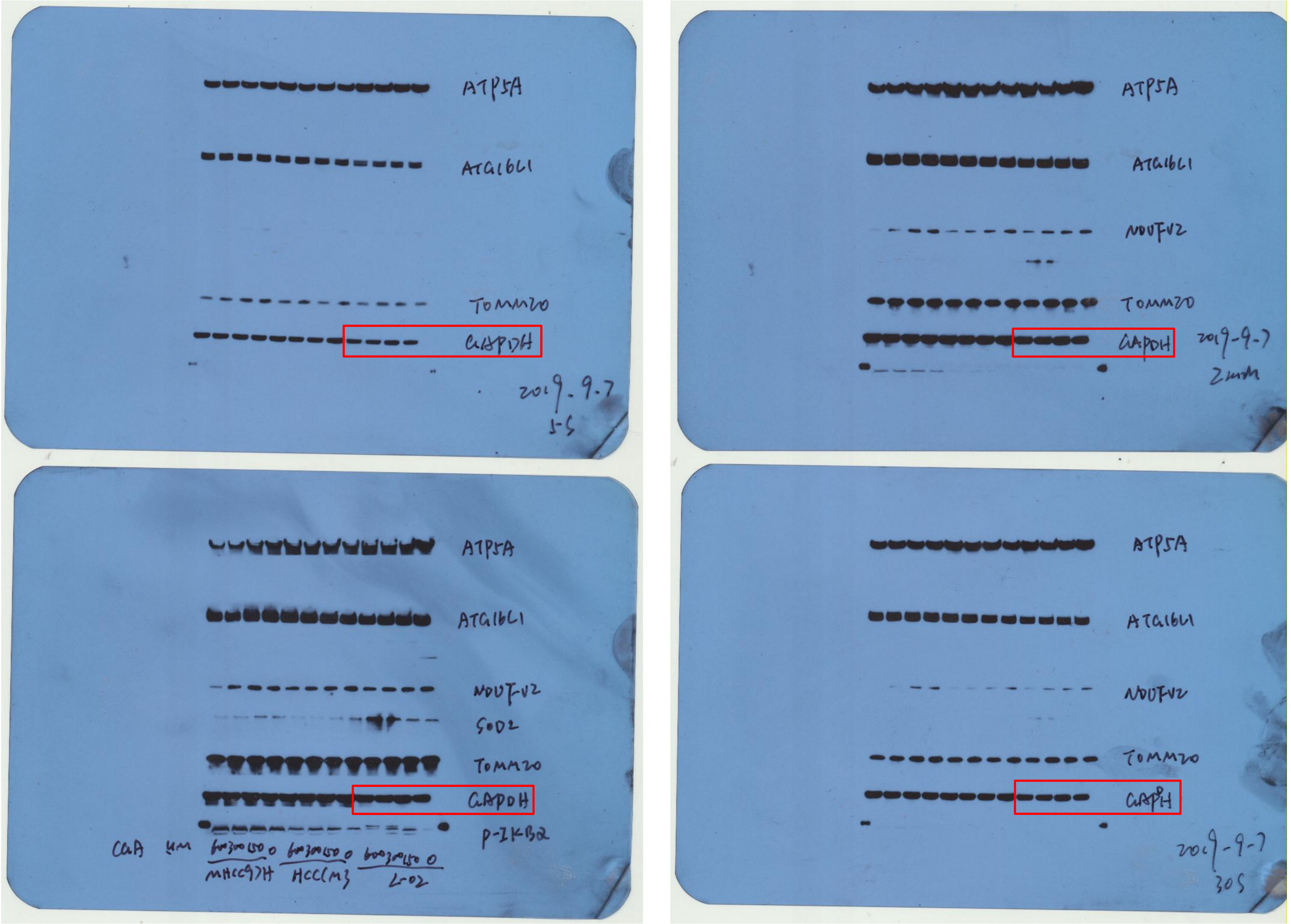

SUPPLEMENTARY FIGURE S7, related to FIGURE 6A. The raw data from the Western blotting experiment.

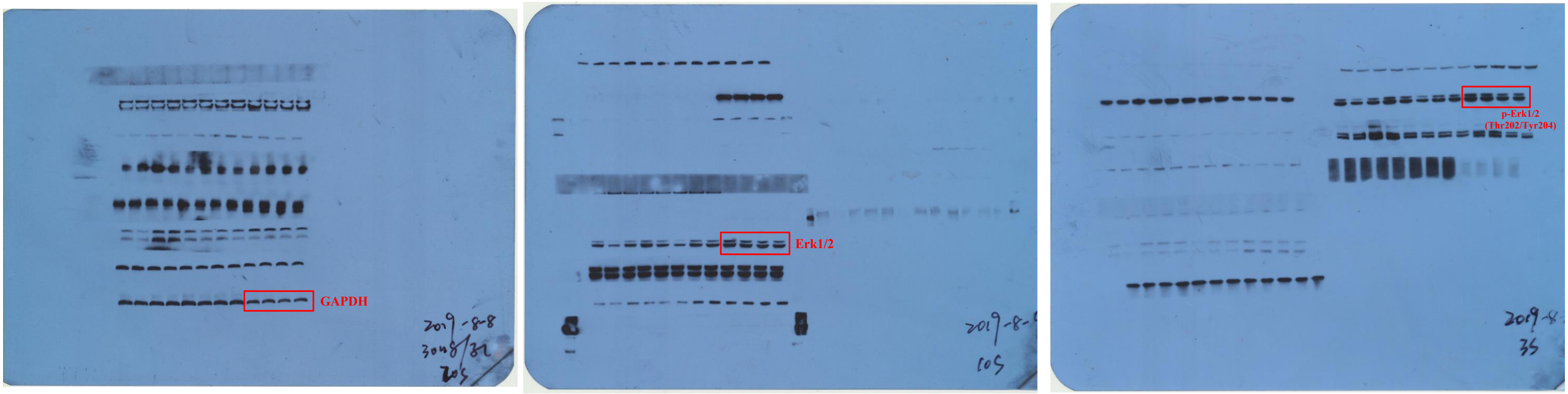

SUPPLEMENTARY FIGURE S8, related to SUPPLEMENTARY FIGURE S3E. The raw data from the Western blotting experiment.

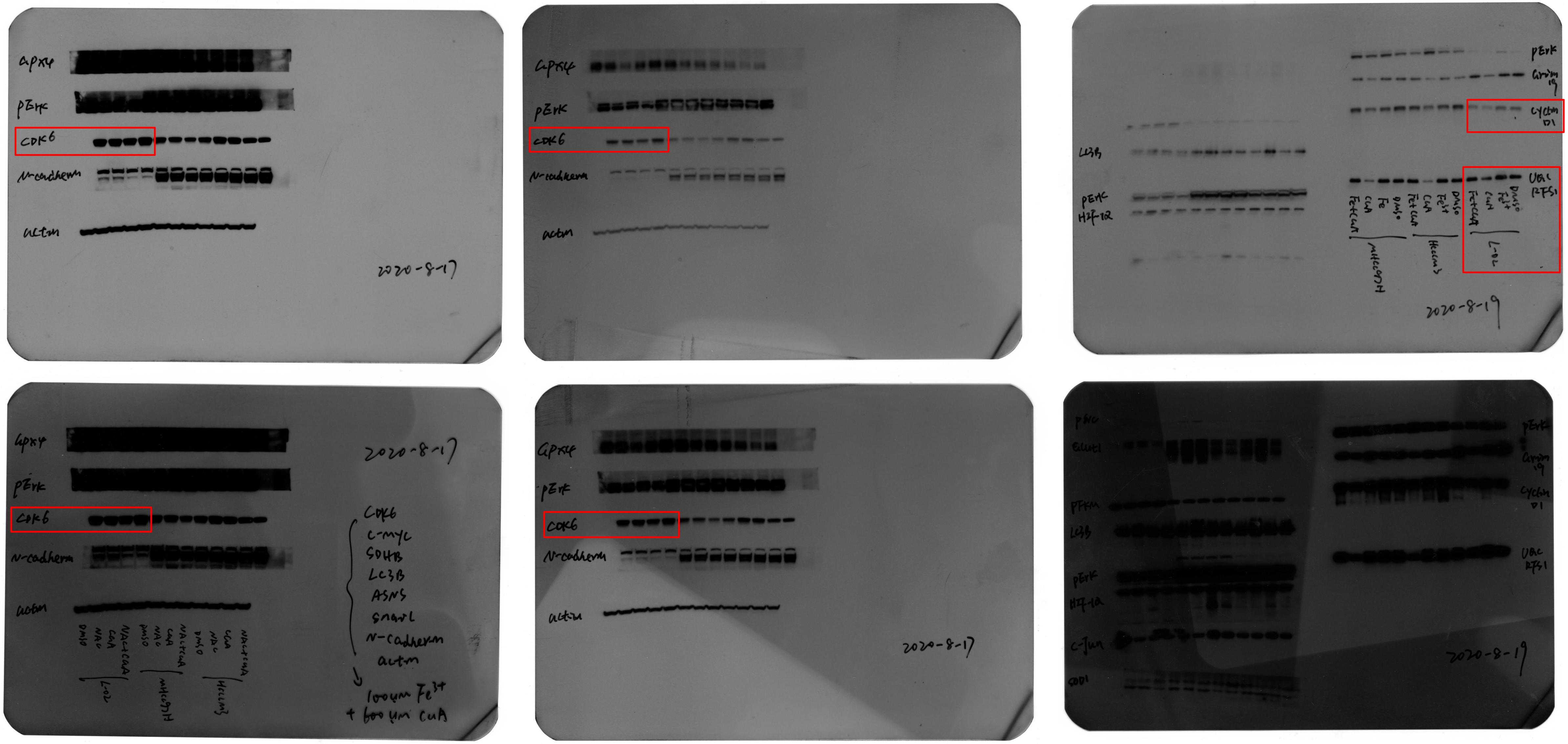

SUPPLEMENTARY FIGURE S9, related to FIGURE 6D. The raw data from the Western blotting experiment.

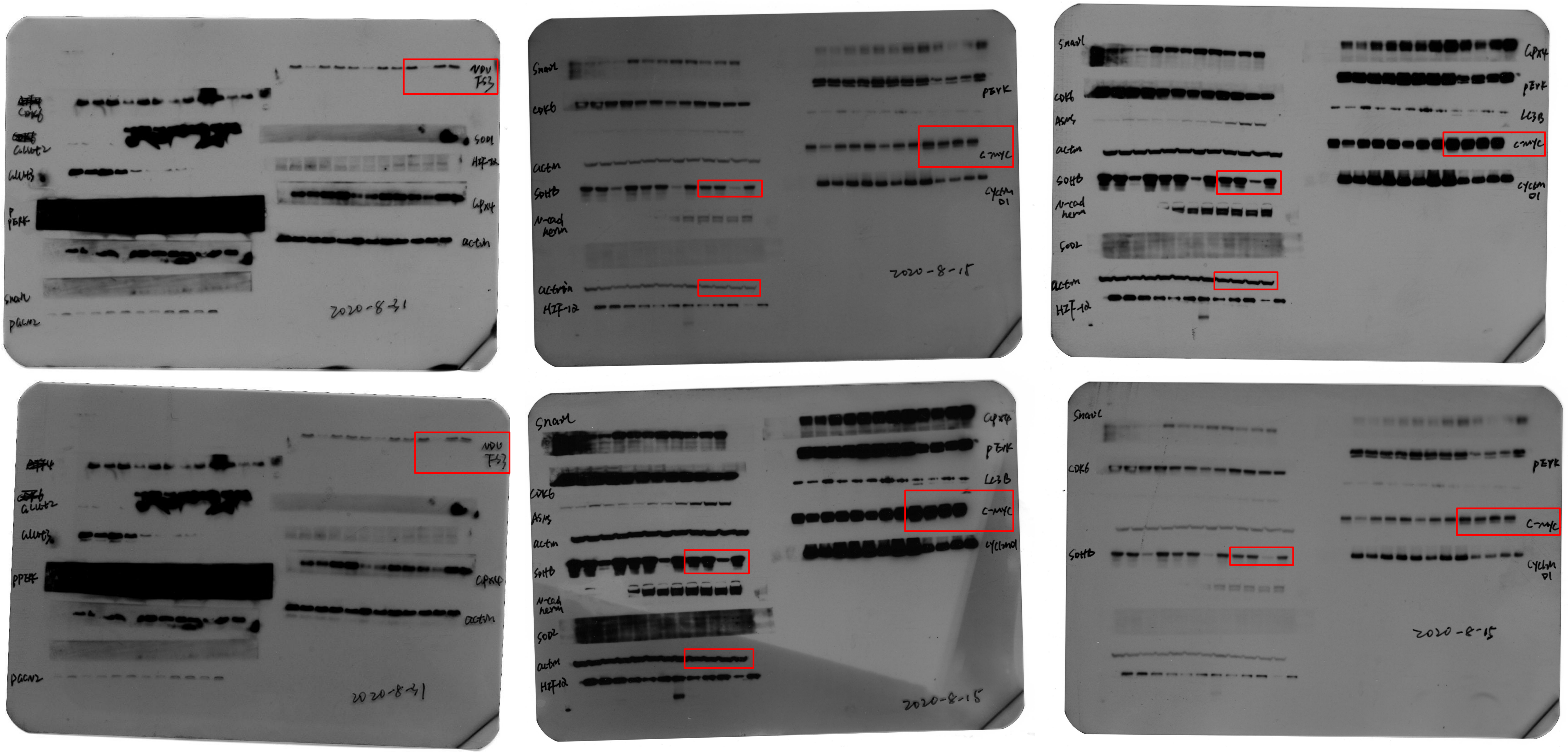

SUPPLEMENTARY FIGURE S9, related to FIGURE 6D. The raw data from the Western blotting experiment.

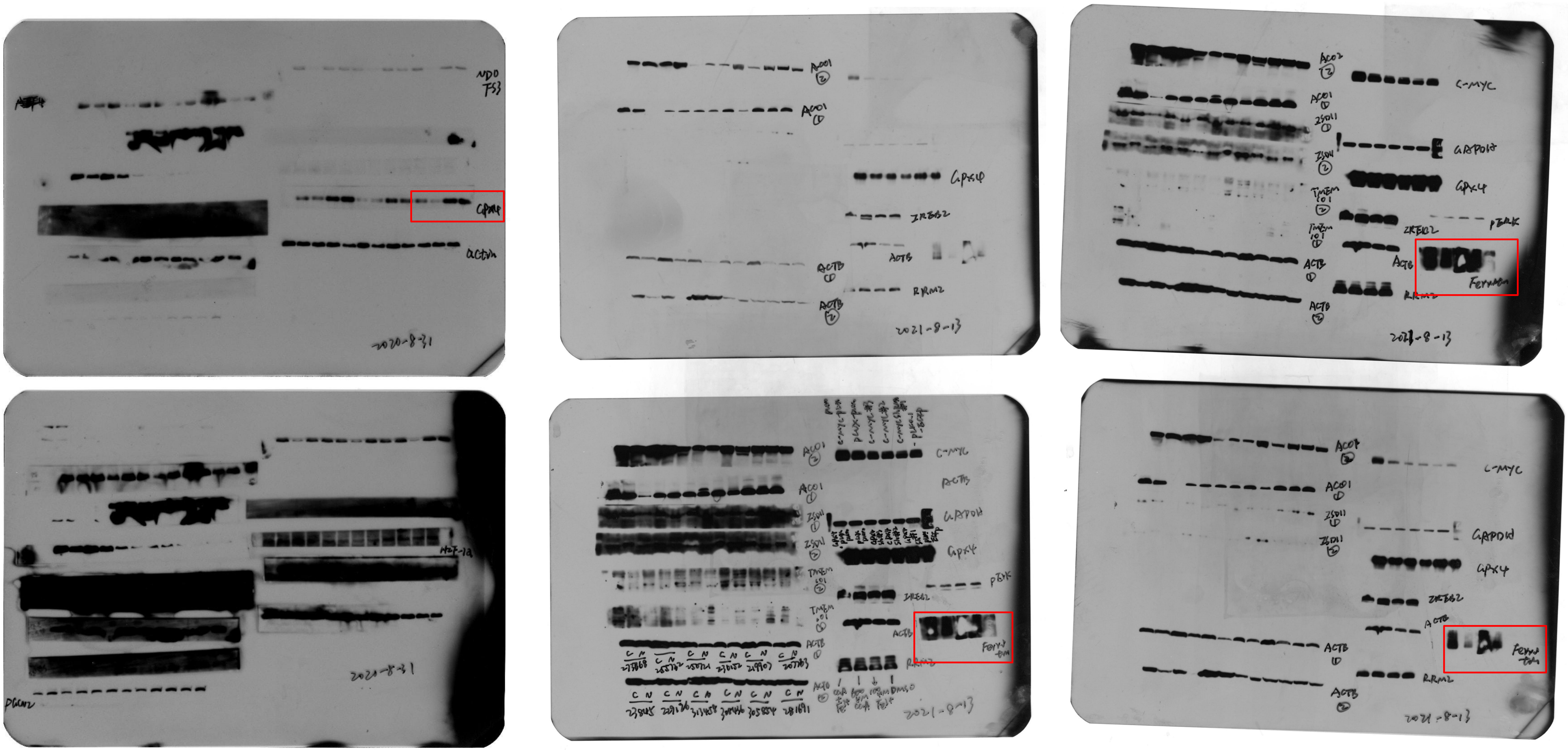

SUPPLEMENTARY FIGURE S9, related to FIGURE 6D. The raw data from the Western blotting experiment.

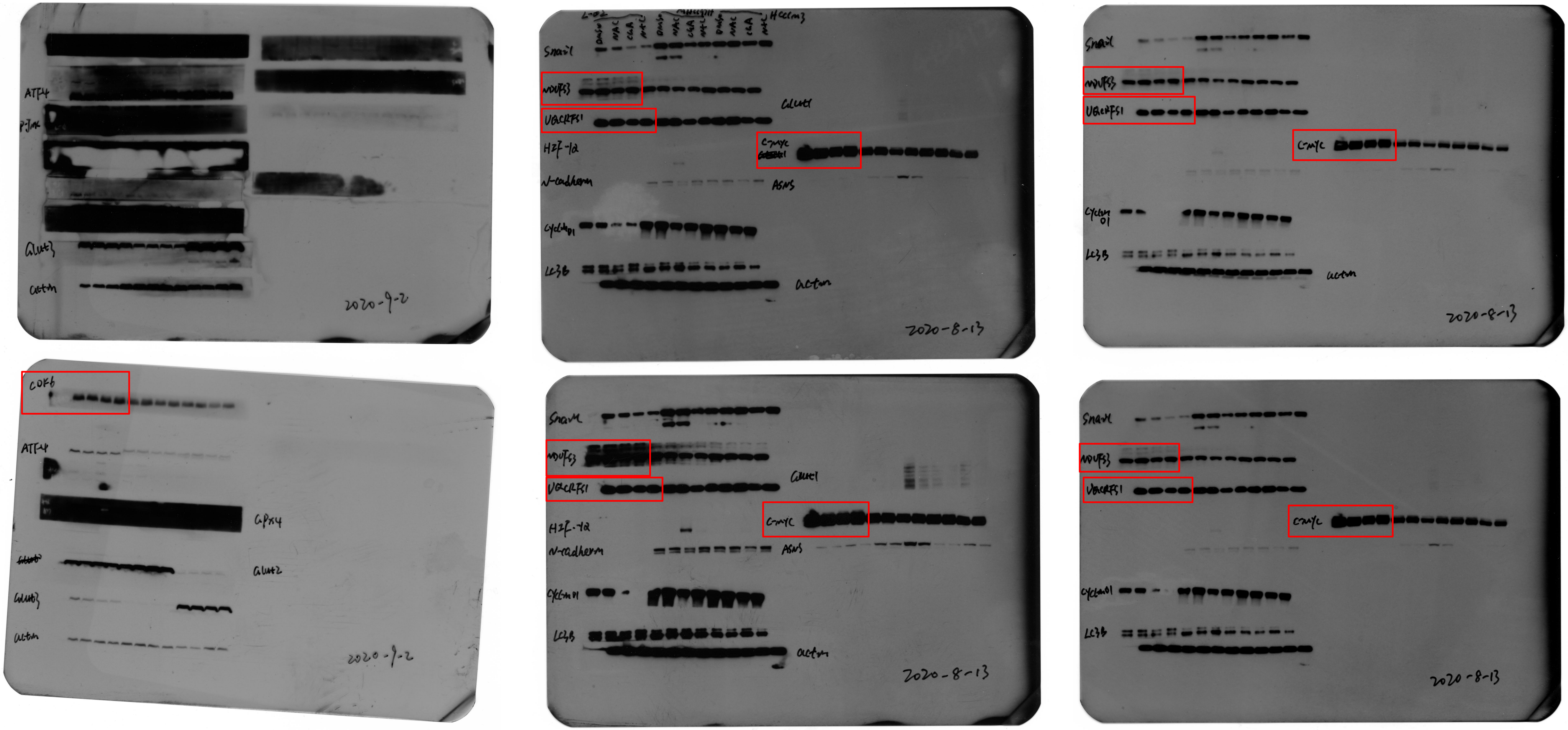

SUPPLEMENTARY FIGURE S10, related to FIGURE 7E. The raw data from the Western blotting experiment.

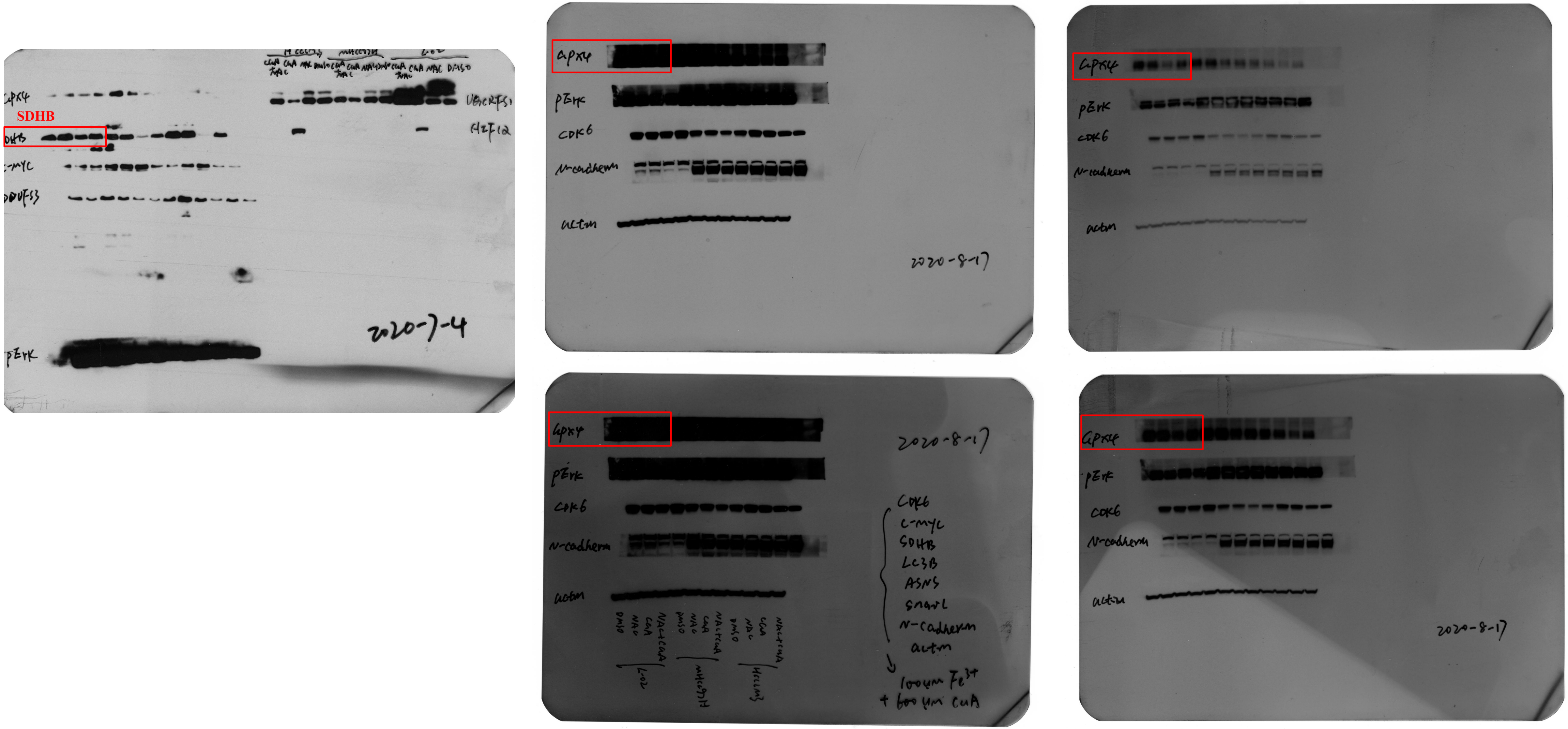

SUPPLEMENTARY FIGURE S10, related to FIGURE 7E. The raw data from the Western blotting experiment.

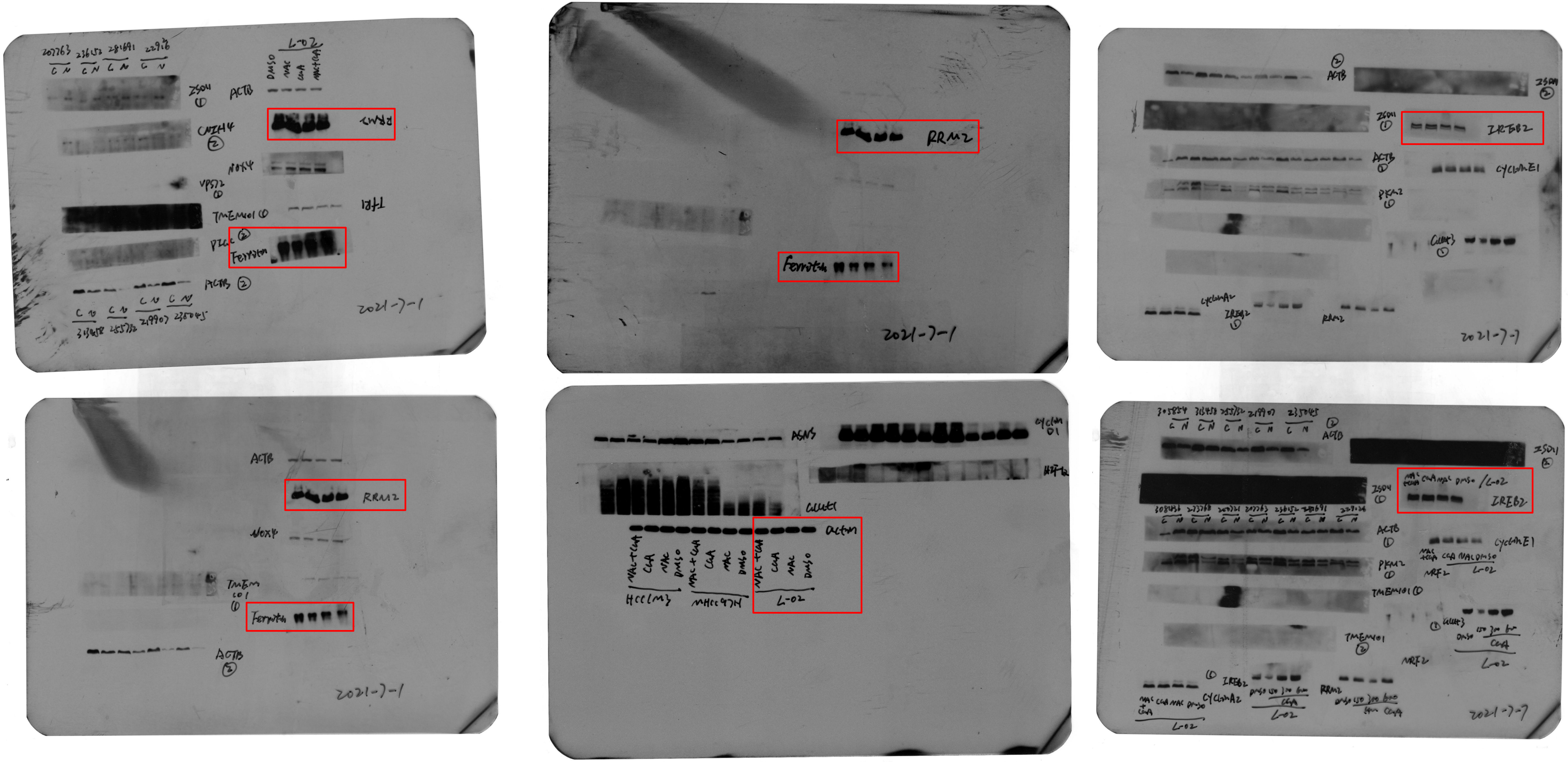

SUPPLEMENTARY FIGURE S10, related to FIGURE 7E. The raw data from the Western blotting experiment.
